# Fabrication of High-Quality Polymer Composite Frame by a New Method of Fiber Winding Process

**DOI:** 10.3390/polym12051037

**Published:** 2020-05-02

**Authors:** Jaroslav Mlýnek, Michal Petrů, Tomáš Martinec, Seyed Saeid Rahimian Koloor

**Affiliations:** 1 Department of Mathematics, FP, Technical University of Liberec, Studentská 2, 461 17 Liberec, Czech Republic; jaroslav.mlynek@tul.cz; 2 Institute for Nanomaterials, Advanced Technologies and Innovation, Technical University of Liberec, Studentská 2, 461 17 Liberec, Czech Republic; michal.petru@tul.cz (M.P.); tomas.martinec@tul.cz (T.M.)

**Keywords:** polymer composite frame, winding of fibers, winding angle, matrix calculus, mathematical model, experimental verification, optimization of robot trajectory

## Abstract

Polymer composite frame has been frequently used in the main structural body of vehicles in aerospace, automotive, etc., applications. Manufacturing of complex curved composite frame suffer from the lack of accurate and optimum method of winding process that lead to preparation of uniform fiber arrangement in critical location of the curved frame. This article deals with the fabrication of high-quality polymer composite frame through an optimal winding of textile fibers onto a non-bearing core frame using a fiber-processing head and an industrial robot. The number of winding layers of fibers and their winding angles are determined based on the operational load on the composite structure. Ensuring the correct winding angles and thus also the homogeneity of fibers in each winding layer can be achieved by using an industrial robot and by definition of its suitable off-line trajectory for the production cycle. Determination of an optimal off-line trajectory of the end-effector of a robot (robot-end-effector (REE)) is important especially in the case of complicated 3D shaped frames. The authors developed their own calculation procedure to determine the optimal REE trajectory in the composite manufacturing process. A mathematical model of the winding process, matrix calculus (particularly matrices of rotations and translations) and an optimization differential evolution algorithm are used during calculation of the optimal REE trajectory. Polymer composites with greater resistance to failure damage (especially against physical destruction) can be produced using the above mentioned procedure. The procedure was successfully tested in an experimental composite laboratory. Two practical examples of optimal trajectory calculation are included in the article. The described optimization algorithm of REE trajectory is completely independent of the industrial robot type and robot software tools used and can also be used in other composite manufacturing technologies.

## 1. Introduction

In the past few decades, polymer composites have increasingly replaced traditional materials such as wood, iron, and steel in advanced industrial applications, due to their superb mechanical features such as flexible design capability, high strength to weight ratio, thermal resistance, etc. [1,2]. Polymer composite frames are mainly used to reinforce the chassis, body, and doors of a car, or to strengthen the fuselage and attach windows to the fuselage, etc., in the aerospace and automotive industries, as well as in the manufacture of agricultural machinery [3,4,5,6]. These composite frames are primarily used due to their excellent mechanical and physical properties such as resistance to harsh weather conditions, as well as long term resistance to corrosion in severe environmental conditions, etc. [7,8,9,10]. Achieving the desired properties of the composite significantly depends on the quality of the wound fibers (usually carbon, aramid, or glass fibers) and the fabrication process [11,12,13]. Ensuring the correct fiber winding angles from the geometric point of view and hence the homogeneity of the windings is one of the important aspects of composite quality. Previous studies indicated that any inhomogeneity during fiber windings process in the fabrication process polymer composite frame, results in preparation of defect as a potential source of defect that induce stress concentration and early failure phenomena [14,15,16,17].

**Figure 1:**
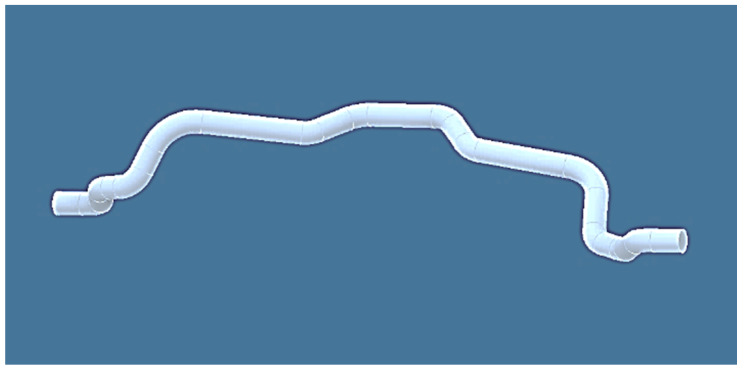
Example of 3D geometrically complicated non-bearing core frame used to reinforce chassis of passenger car.

Polymer composite frames are normally fabricated in complex irregular geometry with various configurations (e.g., Figure 1), in which industrial robots play an important role in the production of the fiber winding process [15]. Once the core frame is wound by the fibers, then it is replaced in a mold where matrix material, such as resin, etc., is injected around the frame to form a solid layer with specific thickness as the polymer composite frame [15,18]. The advantages of robotic fiber over manual fiber windings (winding of fibers by production worker without the use of a robot or other textile machine) in the manufacturing process of composite frame were investigated by Shirinzadeh, et al. [19]. It is proven that determining a correct off-line REE trajectory results in making high-quality winding of fibers onto a core frame during the production process of the polymer composite frame [3,4,16,20]. The process of trajectory calculation during the winding process for simple frame geometry in the form of two- and three-dimensional (2D and 3D) cases, is described elsewhere [21]. However, this trajectory process is not optimized and may not be applicable in the case of a more complicated 3D shaped non-bearing core frame.

Sofi et al. presented dry fiber winding possibilities and explanation of the most important processes of winding [22], and Polini et al. pointed out that the tension of winding during robotic filament winding technology is a very important parameter that influences directly the defects and the mechanical property of composite [23]. Azevedo et al. investigated the effects of mosaic winding pattern on carbon- fiber composite cylinder fabricated using filament winding, and found the optimum radius to thickness ratio for optimum strength and stiffness properties [24]. Many authors studied the problem of the correct winding of fibers on a non-bearing core frame. This problematic field is very topical and essential for the industrial production of composite frames. Along with specific selections of layers sequence and angle that is made through design process, the “correct winding angle process” is to ensure the arrangement of the fibers such that the angle of the fibers to the frame remains uniform, homogeneous, and consistent in circular-helix cross-sectional form throughout the complex 3D curved geometry of the frame. Determination of the correct trajectory of a robot during winding of the fibers is presented, for example, in [19,25,26,27]. Gao et al. proposed high-speed fiber placement technology in a new methodology of motion planning in a redundant robotic system [25], in which the problem of time optimization of robot motion is solved [25,28,29]. The optimization of robot trajectory in the fiber winding process is also addressed, in which graph theory is used to obtain the optimal robot trajectory as well as special algorithm such as genetic algorithm, harmony search and also Bézier curves [28,29,30,31,32]. A similar topic, however, focused rather on the study of trajectory control of an arbitrary shape winding mandrel in 3D circular braiding is explored in [20,23,27].

The present study focuses on the production of high-quality polymer composite frame by calculation of the correct winding angles. In this respect, the winding technology, individual components used in the winding process and the mathematical model of the winding process are described in detail to address the use of a mathematical model, matrix calculus and optimization process. A 3D complex frame is considered for calculating the optimal 3D REE trajectory, as shown in Figure 1. The attempt is to find the correct winding angles and appropriate homogeneous distribution of the fiber windings through this optimization procedure, even in the case of a highly complicated frame geometry. It should be emphasized that this study deals with the issue of defining a suitable cost function in order to find the optimal off-line REE trajectory during individual steps of the frame’s passage through the fiber-processing head to ensure the correct winding of the fibers onto the frame. A differential evolution algorithm is used to obtain a minimum cost function as well as finding the optimal REE trajectory. The possibility of frame collisions with a fiber-processing head is also tested during each passage step. The procedure described in this paper allows ensuring that the geometrically correct required winding angles are maintained during the winding process.

## 2. Manufacturing of Polymer Composite Frame

Technology of polymer composite frame production is a complex process, in which the shape and function of the composite frame (open (Figure 1) or closed) are the determining parameters for the choice of material and technology of the composite parts [33]. The choice of fibrous material depends on the required physical and mechanical properties of final product design based on specific operational load and boundary condition. It is possible to use dry fibers of carbon, glass, basalt, aramid, a combination of these fibers so-called hybrids or a combination these fibers with thermoplastic fibers. The aim of winding of dry fibers is to create directionally oriented layers of fibers so that the fibers are wound homogeneous, regularly, and evenly in each layer. The number of winding layers and the angular orientation of the individual winding fiber layers are usually determined using mathematical models and simulation of the composite frame [1,33,34,35,36,37]. The winding process is generally done through “manual-robotic winding” in which the robot is programmed manually (teach-in method). This method is time-consuming and does not guarantee a quality winding process for more geometrically complex frame shape.

**Figure 2:**
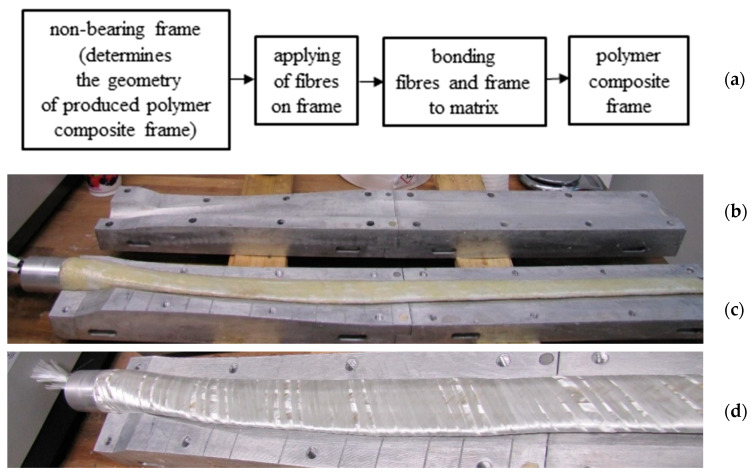
(**a**) production process of long-fiber reinforced polymer composite frame, (**b**) a laboratory mold for holding and curing of the polymer composite frame (example of wind turbine blade), (**c**) the core before fiber winding, and (**d**) wound core with fibers, positioned in the mold before injection of the polymer matrix.

There are a few procedures for applying the fiber reinforcement to the core frame, such as methods for overlaying the fibers on the core frame, and winding the fibers from the coils while rotating the core frame [1]. Most of the fiber winding procedures are applicable to constant fiber deposition without possible optimization of the fiber laying angles in 3D. The procedure described in this article allows to optimize geometrically fiber winding on 3D open and closed frames, in which the production process is shown in Figure 2a. In the first step of the production of a composite frame, a mold (Figure 2b) with the geometry identical to the final product is fabricated to make a non-bearing and lightweight core frame (generally made of porous polyurethane foam, brown color frame in Figure 2c). Then, the fiber is wound around the core frame (Figure 2d), and the frame is inserted into the preheated mold and then the matrix (made of thermoplastics (polyurethane, etc.) or thermosets (epoxy, polyester, etc.) are injected into the mold under controlled pressure and temperature for the curing process as dictated through the vacuum injection technology.

### 2.1. Fiber Winding Geometry

This section describes a geometric interpretation of the execution of the fiber winding on a non-bearing core frame of circular cross-section.

The right-handed Euclidean coordinate system *E*_3_ is taken into account. Vectors and matrices are written in a homogeneous form (i.e., any point $V={\left[x_{V},y_{V},z_{V},1\right]}^{T}$ and any vector $u={\left(x_{u},y_{u},z_{u},0\right)}^{T}$, in more detail see [38]). The Euclidean norm ‖u‖ of vector u, where $\left\|u\right\|=\sqrt{x_{u}^{2}+y_{u}^{2}+z_{u}^{2}}$ is used.

**Figure 3:**
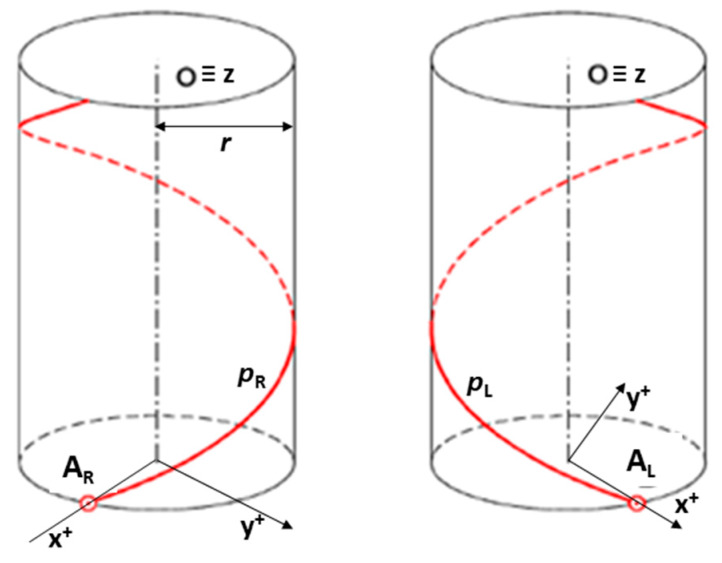
Right-handed helix $p_{R}$ with initial point $A_{R}$ and left-handed helix $p_{L}$ with initial point $A_{L}$.

The winding of one fiber on a composite frame of a circular cross-section is from a geometrical point of view generally a helical motion. The winding fiber creates the helix on the composite frame. This motion is the composition of rotation of a fiber around the internal axis o of the frame and its parallel translation in direction of axis o. In this way, fiber forms a helix on the surface of the frame. Right-handed and left-handed helices are shown in Figure 3. Points $A_{R}$ and $A_{L}$ are the initial points of helices created by the winding process.

Let us consider the right-handed helix $p_{R}$ with axis o identical to axis z of the right-handed Euclidean coordinate system $E_{3}$ (Figure 3 (left)) and its parameters reduced pitch $v_{0}$ (length of translation during rotation of fiber by one radian), helix radius r (radius of frame), angle δ of slope of the helix $p_{R}$ defined by relation $tg\delta =v_{0}/r$ (the detailed description of helix parameters can be found elsewhere [39,40]). Then the parametric equation of helix $p_{R}$ can be expressed in the form of

$$p_{R}(t)=\left(r\;\cos t,\;r\;\sin t,\;v_{0}t,\;1\right),\;t\in <\;0,\;\infty )$$

Point $A_{R}={\left(r,\;0,\;0,\;1\right)}^{T}$ is then the initial point of right-handed helix $p_{R}$.

The equation of the right-handed helix $p_{R}$ can also be expressed as the rotation of point $A_{R}={\left(r,\;0,\;0,\;1\right)}^{T}$ around axis z (we suppose o≡z) and its translation in a positive direction of axis z [39]:

(1) $$p(t)={\left(x(t),\;y(t),\;z(t),\;1\right)}^{T}=\left[\begin{array}{cccc} \cos t & -\sin t & 0 & 0\\ \sin t & \cos t & 0 & 0\\ 0 & 0 & 1 & 0\\ 0 & 0 & 0 & 1 \end{array}\right]\cdot \left[\begin{array}{cccc} 1 & 0 & 0 & 0\\ 0 & 1 & 0 & 0\\ 0 & 0 & 1 & v_{0}t\\ 0 & 0 & 0 & 1 \end{array}\right]\cdot \left[\begin{array}{c} r\\ 0\\ 0\\ 1 \end{array}\right]=\;\left[\begin{array}{cccc} \cos t & -\sin t & 0 & 0\\ \sin t & \cos t & 0 & 0\\ 0 & 0 & 1 & v_{0}t\\ 0 & 0 & 0 & 1 \end{array}\right]\cdot \left[\begin{array}{c} r\\ 0\\ 0\\ 1 \end{array}\right]=\left[\begin{array}{c} r\cos t\\ r\sin t\\ v_{0}t\\ 1 \end{array}\right].$$

**Figure 4:**
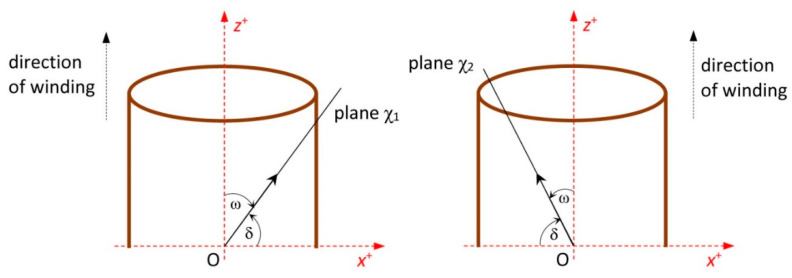
Scheme—front view of the non-bearing core frame with (**left**) positive and (**right**) negative angle of fiber winding on the frame. Winding angle ω is angle between central axis z of the frame and plane of fiber winding $\chi_{1}$ (respectively $\chi_{2}$).

In the case of one turn of the right-handed helix $p_{R}$ as shown in Figure 3 (left), parameter t lies within interval t∈<0, 2π>. Please note that the positive winding angle ω of one fiber on composite frame is (Figure 4 (left))

(2) $$\omega =\frac{\pi }{2}-\delta$$

and if the value of reduced pitch $v_{0}$ is close to ∞, then ω=0 (in this case the fiber is laid on the frame parallel with internal axis o≡z of the frame).

In the case of winding negative angle ω of one fiber on the composite frame, left-handed helix $p_{L}$ is considered and angle ω is (Figure 4 (right))

(3) $$\omega =-\left(\frac{\pi }{2}-\delta \right)$$

The parametric equation of left-handed helix $p_{L}$ can be expressed as

$$p_{L}(t)=\left(r\;\cos\;t,\;-r\;\sin t,\;v_{0}t,\;1\right),\;t\in <\;0,\;\infty )$$

and similarly by matrix calculus as in the case of the right-handed helix in relation (1).

In the production of the polymer composite frame, three layers of fibers during the winding process and twelve strands of fibers are usually wound on the frame in each layer. One given winding layer with positive winding angle ω is considered. Then during the winding process each strand (of the total number of twelve strands) creates right-handed helix $p_{R}$ on the composite frame with positive angle δ of slope of the helix. In accordance with relation (2), $\omega =\frac{\pi }{2}-\delta$. Let angles $\lambda_{i}$ are determined by relation $\lambda_{i}=(i-1)\;2\pi /12$ for i=1, 2, .…, 12.

**Figure 5:**
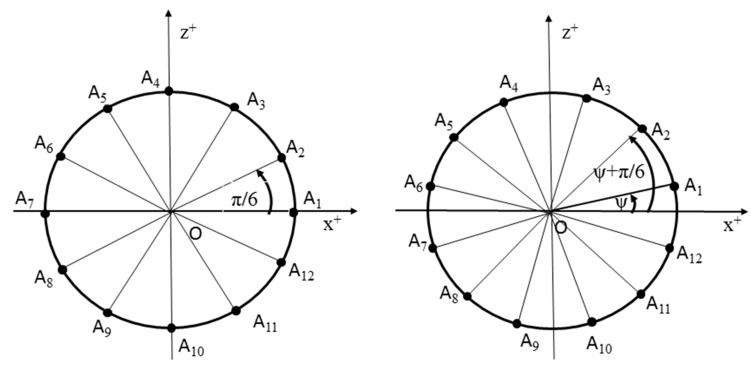
Initial points of the winding right-handed helices $p_{R}{}_{i}$ (**left**) and with rotation angle *ψ* (**right**) of the winding layer.

A circle with its center in origin O of right-handed Euclidean coordinate system *E*_3_ and radius r is considered. Then arms of oriented angles $\lambda_{i}$ create on the circle the vertices $A_{i}={\left(r\cos\lambda_{i},\;r\sin\lambda_{i},\;0,\;1\right)}^{T}$ of a regular twelve-rectangle (Figure 5 (left)).

Vertices $A_{i}$ are initial points of individual helix $p_{R}{}_{i}$ (i=1, 2, .…, 12) of the winding layer. The parametric equation of right-handed helix $p_{R}{}_{i}$ in accordance with relation (1) can be defined in the form

$$p_{R}{}_{i}(t)={\left(x(t),\;y(t),\;z(t),\;1\right)}^{T}=\left[\begin{array}{cccc} \cos t & -\sin t & 0 & 0\\ \sin t & \cos t & 0 & 0\\ 0 & 0 & 1 & 0\\ 0 & 0 & 0 & 1 \end{array}\right]\cdot \left[\begin{array}{cccc} 1 & 0 & 0 & 0\\ 0 & 1 & 0 & 0\\ 0 & 0 & 1 & v_{0}t\\ 0 & 0 & 0 & 1 \end{array}\right]\cdot \left[\begin{array}{c} r\cos\lambda_{i}\\ r\sin\lambda_{i}\\ 0\\ 1 \end{array}\right]=\;=\left[\begin{array}{cccc} \cos t & -\sin t & 0 & 0\\ \sin t & \cos t & 0 & 0\\ 0 & 0 & 1 & v_{0}t\\ 0 & 0 & 0 & 1 \end{array}\right]\cdot \left[\begin{array}{c} r\cos\lambda_{i}\\ r\sin\lambda_{i}\\ 0\\ 1 \end{array}\right]=\left[\begin{array}{c} r\cos t\cos\lambda_{i}-r\sin t\sin\lambda_{i}\\ r\sin t\cos\lambda_{i}+r\cos t\sin\lambda_{i}\\ v_{0}t\\ 1 \end{array}\right]=\left[\begin{array}{c} r\cos(t+\lambda_{i})\\ r\sin(t+\lambda_{i})\\ v_{0}t\\ 1 \end{array}\right]$$

Helix $p_{R}{}_{i}(t)={\left(r\cos(\;t+\lambda_{i}),\;r\sin(t+\lambda_{i}),\;v_{0}t,\;1\right)}^{T}$ can be obtained by this way of expression. If the considerations are generalized and the rotation of points $A_{i}$ around axis z of the coordinate system is considered, (Figure 5 (right)) with angle ψ, the expression of helix $p_{R}{}_{i}$ can be written in the form

(4) $$p_{R}{}_{i}(t)={\left(r\cos(\psi +t+\lambda_{i}),\;r\sin(\psi +t+\lambda_{i}),\;v_{0}t,\;1\right)}^{T}\;\mathrm{for}\;i=1,\;2,\;.\ldots ,\;12;\;t\in <\;0,\;\infty )$$

Individual helices $p_{R}{}_{i}$ of the winding layer have the same winding angle ω, but the next helix is rotated by angle $\frac{\pi }{6}$ relative to the previous one.

The generalized parametric equation of the left-handed winding helix in the form (4) can be derived analogously.

All layers of windings (with positive, negative or zero winding angle ω) are progressively realized along the entire circumference of the non-bearing core frame in the case of the composite frame. In practical cases the composite frame can be open or closed.

### 2.2. Mathematical Model of Winding Process

**Figure 6:**
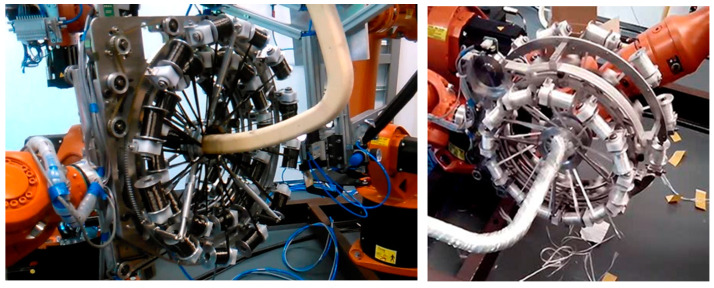
Different views of robot KR 16-2 with non-bearing core frame and fiber-processing head with three guide lines.

A brief description of the mathematical model of the winding process and defined designations and abbreviations are given in this section The actual process of winding the fibers on a non-bearing frame is implemented by the fiber-processing head (Figure 6 (right)) and by an industrial robot (in this article industrial robot KUKA KR 16-2).

**Figure 7:**
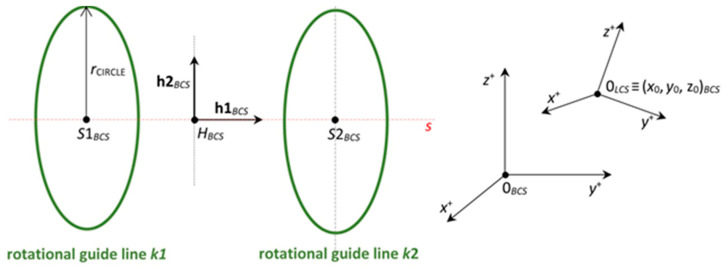
Fiber-processing head in mathematical model in BCS (**left**) and coordinate systems BCS and LCS (**right**).

The non-bearing core frame is firmly attached to the REE, and the fiber-processing head is fixed in the workspace of the robot (Figure 6 (left)). In the described mathematical model, the basic right-handed Euclidean coordinate system *E*_3_ of the robot (BCS) is taken into account. This system is often called the “robot coordinate system” for industrial robots (Figure 7 (right)). Individual parts of the mathematical winding model are described in BCS.

The local right-handed Euclidean coordinate system *E*_3_ of the REE (LCS) is also taken into account. The position of LCS toward BCS defines position of REE in BCS.

The points and vectors with coordinates in BCS and LCS are labeled with the subscript ${}_{BCS}$ and ${}_{LCS}$ in the following text, respectively.

#### 2.2.1. Fiber-Processing Head

The visual presentation of the fiber-processing head (Figure 6) in the mathematical model is given in Figure 7 (left). The coordinates of the head individual components are defined in the BCS. The first outer rotating guide line with fiber spools forms the first winding layer of fibers (usually under the angle 45°, see Figure 4 (left)) and is presented by circle k1 with center $S1_{BCS}=\left[x_{S1}{}_{BCS},y_{S1}{}_{BCS},z_{S1}{}_{BCS},\;1\right]$. The second guide line creates a layer of longitudinally laid fibers (winding under angle 0°—parallel to the frame axes $o_{BCS}$) which is not important in the mathematical model. The third guide line forms the last layer of fibers (usually under the angle—45°) and is presented by circle k2 with center $S2_{BCS}={\left[x_{S2}{}_{BCS},y_{S2}{}_{BCS},z_{S2}{}_{BCS},\;1\right]}_{}$. Circles k1 and k2 have the same radius $r_{\mathrm{CIRCLE}}.$ Wounded fibers by circles (guide lines) k1 and k2 on the frame create a right and left-handed helix (Figure 3). Points $S1_{BCS}$ and $S2_{BCS}$ lie on axis $s_{BCS}$ of the fiber-processing head (Figure 7 (left)). Axis $s_{BCS}$ is parallel to the coordinate axis $y_{BCS}$ of system BCS ($s_{BCS}//y_{BCS}$) in the model.

#### 2.2.2. Non-Bearing Core Frame

**Figure 8:**
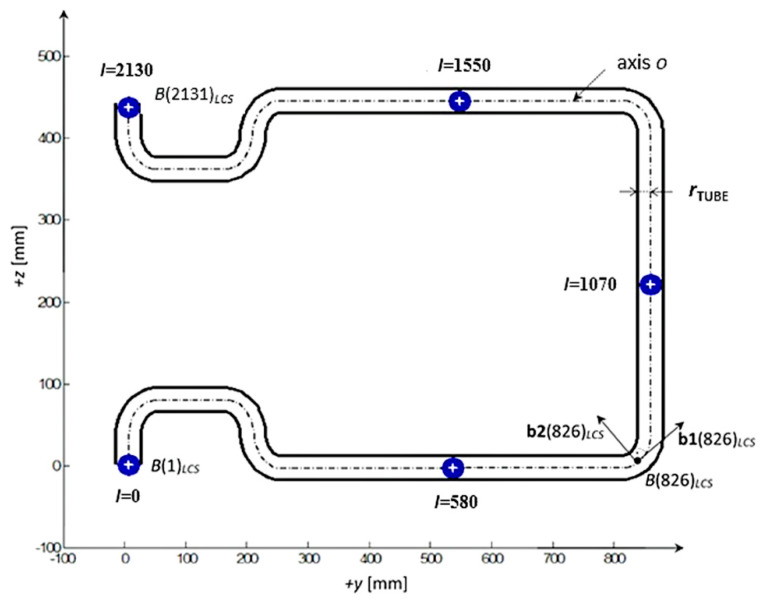
Example of a vertical cross-section of a non-bearing core frame with axis o in LCS—frame for a baby carriage. The frame is connected to the REE at the point $B{\left(N\right)}_{LCS}$, where N=2131. Unit tangent vector $b1{\left(i\right)}_{LCS}$ to axis $o_{LCS}$ and unit vector $b2{\left(i\right)}_{LCS}$ are defined at point $B{\left(i\right)}_{LCS}$ for i= 1, … ,N. All the time $b1{\left(i\right)}_{LCS}⊥b2{\left(i\right)}_{LCS}$ holds (vector $b2{\left(i\right)}_{LCS}$ characterizes the needed rotation of the frame around axis $o_{LCS}$ when point $B{\left(i\right)}_{LCS}\in o_{LCS}$ passes through fiber-processing head).

The non-bearing core frame with a circular cross-section is defined by central axis $o_{LCS}$ and radius $r_{TUBE}$ (see example of frame in Figure 8). We suppose $r_{\mathrm{CIRCLE}}>r_{TUBE}$. Central axis $o_{LCS}$ is entered in LCS of the REE using a discrete set of N points $B{\left(i\right)}_{LCS}$ lying on axis $o_{LCS}$, where 1≤i≤N. The initial point of axis $o_{LCS}$ is $B{\left(1\right)}_{LCS}$ and endpoint is $B{\left(N\right)}_{LCS}$. At the same time, for each index i unit tangent vector $b1{\left(i\right)}_{LCS}$ to axis $o_{LCS}$ and unit vector $b2{\left(i\right)}_{LCS}$ at point $B(i){}_{LCS}$ are defined. All the time $b1(i){}_{LCS}⊥b2{(i)}_{\;LCS}$ holds (see Figure 8). Vector $b2\;{\left(i\right)}_{LCS}$ characterizes the needed rotation of the frame around axis $o_{LCS}$ when frame passes through fiber-processing head (detail is described elsewhere [21]). In the case of a closed frame $B{\left(1\right)}_{\;LCS}\equiv B{\left(N\right)}_{\;LCS},$ $b1\;{(1)}_{LCS}\equiv b1\;{\left(N\right)}_{LCS}$ and $b2\;{\left(N\right)}_{LCS}$ apply.

The variable l (see Figure 8) represents the distance of a general point lying on the axis $o_{LCS}$ from point $B{\left(1\right)}_{LCS}$ measured on the axis $o_{LCS}$ (point $B{\left(1\right)}_{LCS}$ is at the beginning of the frame, this distance l is marked as the o-arc length). Points of axis $o_{LCS}$ with increasing distance l gradually pass through the head at the winding process. The set of positive real non-negative values C(i) (for 1≤i≤N) is supposed, where value C(i) indicates the o-arc length of point $B{\left(i\right)}_{LCS}$ from point $B{\left(1\right)}_{LCS}$, i.e., $C(i)=l_{B{\left(i\right)}_{LCS}}$. Then value C(N) indicates the total length of the frame.

During the passage of the frame through the fiber-processing head, three layers of fiber windings are created. These layers are created gradually from the beginning of the frame determined by the initial point $B{\left(1\right)}_{LCS}$ of axis o to endpoint $B{\left(N\right)}_{LCS}$ of axis $o_{LCS}$.

An important assumption of the mathematical model is to ensure a constant ration of the rotational angular speed of guide line of the fiber-processing head and speed of passage of the frame through the fiber-processing head. Robot external axes can be used to control rotational angular speed of the outer guide lines *k*1 and *k*2.

The actual position of the LCS of the REE with regard to the BCS is determined by six parameters listed in the “tool-center-point” (TCP). The first three values of TCP=(x,y,z,a,b,c) specify the coordinates of the origin of the LCS in regard to the BCS (see Figure 7 on the right). The last three parameters a, b and c determine the angles of the rotations of the LCS around the axis z, y, and x with regard to the BCS.

### 2.3. Robot Trajectory Optimization

Robot trajectory optimization is usually required for more geometrically 2D and 3D shaped non-bearing core frames. The procedure for optimal robot trajectory calculation is described in this section.

**Figure 9:**
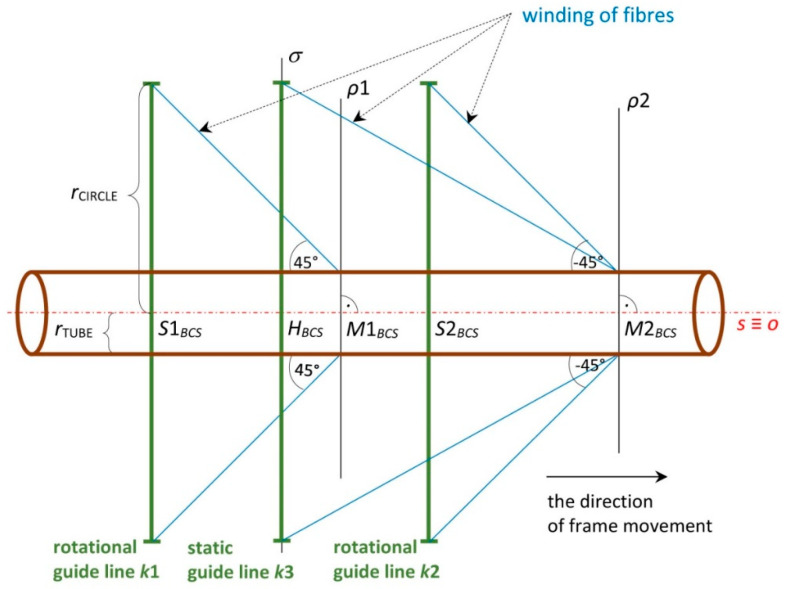
Schematic front view of winding fiber layers onto the non-bearing core frame, case when axis $S_{BCS}$ of the fiber-processing head and axis $O_{BCS}$ of the frame are identical in displayed section, axis $S_{BCS}$ is parallel to the coordinate axis $y_{BCS}$.

**Figure 10:**
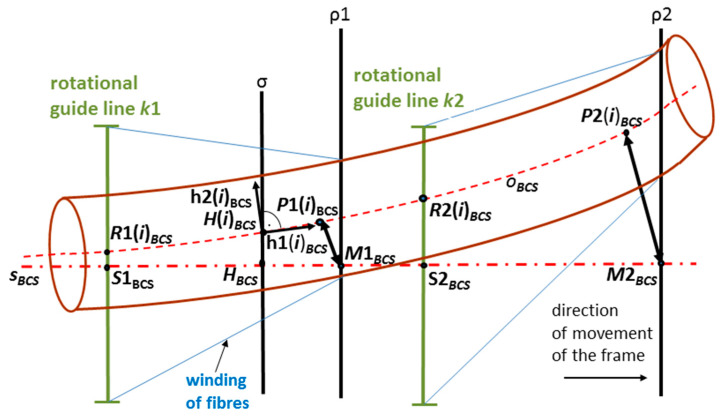
Front view of general scheme of non-bearing core frame passage through the fiber-processing head, axis $s_{BCS}$ of fiber-processing head is parallel to axis $y_{BCS}$.

The winding of three layers of fibers is carried out in the production of the composite frame. Three consecutive layers of fibers are often wound onto the frame at angles of $45^{\circ }$, $0^{\circ }$ and $-45^{\circ }$ (Figure 6). Middle longitudinal layer of fibers is fastened to the frame by the third fiber winding. Please note that in the considered model, axis $s_{BCS}$ of the fiber-processing head is parallel to the coordinate axis $y_{BCS}$. The correct winding angle and homogeneity of the winding fibers (filaments) on the frame are assured if the frame axis $o_{BCS}$ passes through a fictitious winding plane at the same point as the head axis $s_{BCS}$ and at the same time the axis $o_{BCS}$ is orthogonal to the winding plane at that point. The fictitious plane of the first winding layer is designated ρ1 and the fictitious plane of the third winding layer is designated ρ2, as shown in Figure 9 and Figure 10.

The exact winding angles are ensured when frame axis $o_{BCS}$ (or its part) is identical to axis $s_{BCS}$ of fiber-processing head in BCS during winding process (i.e., the frame or its part is a straight rod). Then axis $o_{BCS}$ is orthogonal to the imaginary planes of the fibers winding ρ1 and ρ2 (Figure 9) in points of intersections $M1_{BCS}\in s_{BCS}$ and $M2_{BCS}\in s_{BCS}.$ Static guide line k3 creates the middle layer of longitudinally laid fibers.

The general scheme of the frame passage through the head is displayed in Figure 10. In this case, the axes $o_{BCS}$ and $s_{BCS}$ occupy different positions in the BCS.

The attempt is to optimize the position of the frame when passing through the winding planes ρ1 and ρ2 so that its position would be as close as possible to the case of a straight rod. We are gradually looking for an optimal frame passage through the fiber-processing head for N points $B{\left(i\right)}_{lCS}\in o_{LCS}$ (lying on the *o*-axis) in the time, when point $B{\left(i\right)}_{BCS}\in \sigma$ (where σ is orthogonal plane to axes $s_{BCS}$)—we speak about the *i*-th step of passage of frame through the fiber-processing head, the center of fiber-processing head is point $H_{BCS}\in \sigma$ (Figure 10).

Points $P1\;{\left(i\right)}_{BCS}\in o_{BCS}$ and $P2\;{\left(i\right)}_{BCS}\in o_{BCS}$ are selected to meet the following conditions. The distance of points $R1{\left(i\right)}_{BCS}\in k1$ (this point lies inside k1 in the intersection with axis $o_{BCS}$) and $P1\;{\left(i\right)}_{BCS}\in o_{BCS}$ measured as o-arc length (distance of these points on axis $o_{BCS}$, see Section 2.2.2) is equal to value $\left\|S1_{BCS}M1_{BCS}\right\|$. At the same time, distance points $R2{\left(i\right)}_{BCS}\in k2$ (this point lies inside k2 in the intersection with axis $o_{BCS}$) and $P2{\left(i\right)}_{BCS}\in o_{BCS}$ measured as o-arc length is equal to value $\left\|S2_{BCS}M2_{BCS}\right\|$. The prescribed angles of the first and third windings of the fibers will be more accurate, the smaller the distances ${\left\|P1{\left(i\right)}_{BCS}M1_{BCS}\right\|}^{}$ and $\left\|P2{\left(i\right)}_{BCS}M2_{BCS}\right\|$ will be in the *i*-th step of the frame passage through the winding head. We find such a position of the REE for each i (1≤i≤N) that the location of axis $o_{BCS}$ in BCS (and hence the frame) will make the distances ${\left\|P1{\left(i\right)}_{BCS}M1_{BCS}\right\|}^{}$ and $\left\|P2{\left(i\right)}_{BCS}M2_{BCS}\right\|$ as small as possible (in the case that the frame or its part is a straight rod both distances are equal to zero).

Using the mathematical fiber winding model described in Section 3, matrix calculus and the optimization method, the optimal trajectory of the REE is calculated, which means the optimal passage of the frame through the winding head. In this way, we ensure the correct angle of winding individual layers.

It should be highlighted that the procedure for calculating the optimal trajectory of the REE in the *i*-th step of the frame passage through fiber-processing head, is described in this section.

Point $H_{BCS}\equiv (S1_{BCS}+S2_{BCS})/2$ is the center of the fiber-processing head (Figure 7 (left)) and $h1_{BCS}=(0,\;1,\;0,\;0)$, $h2_{BCS}=(0,\;0,\;1,\;0)$ are constant mutually orthogonal vectors.

Plane σ is orthogonal to axis $s_{BCS}$ of the fiber-processing head (and hence also to axis $y_{BCS}$) and passes through the center $H_{BCS}$ of the head (Figure 9 and Figure 10). Point $H{\left(i\right)}_{BCS}$ is randomly chosen in a defined circle with center $H_{BCS}$, circle lies in plane σ. At the same time, a pair of orthogonal vectors $h1\;{\left(i\right)}_{BCS}$ and $h2\;{\left(i\right)}_{BCS}$ is created through the following relation

(5) $$h1\;{\left(i\right)}_{BCS}=Rot\;(z,\varphi \;(i))\cdot Rot\;(x,\omega \;(i))\cdot h1_{BCS},\;h2{\left(i\right)}_{BCS}=Rot\;(z,\varphi \;(i))\cdot Rot\;(x,\omega \;(i))\cdot h2_{BCS},$$

where Rot(z,φ(i)) is an orthogonal rotation matrix around axis z at angle φ(i) and Rot (x,ω (i)) is an orthogonal rotation matrix around axis x at angle ω (i). Elements of matrices Rot (z,φ (i)) and Rot(x,ω(i)) are listed elsewhere [21,38]. In relation (5) vector $h1_{BCS}$ is first rotated around the *z*-axis by angle φ(i) and then around the x-axis by angle ω (i) and vector $h1\;{\left(i\right)}_{BCS}$ is obtained. The sizes of angles φ (i) and ω (i) are randomly chosen and are within the limits set. Analogously vector $h2\;{\left(i\right)}_{BCS}$ is constructed in relation (5).

In this way we randomly chose point $H{\left(i\right)}_{BCS}\in \sigma$ and mutually orthogonal vectors $h1\;{\left(i\right)}_{BCS}$ and $h2\;{\left(i\right)}_{BCS}$. Then there is an unambiguously defined transformation matrix T(i) that is valid

(6) $$H{\left(i\right)}_{BCS}\equiv B{\left(i\right)}_{BCS}=T(i)B{\left(i\right)}_{LCS},h1{\left(i\right)}_{BCS}\equiv b1\;{\left(i\right)}_{BCS}=T(i)b1\;{\left(i\right)}_{LCS},h2{\left(i\right)}_{BCS}\equiv b2\;{\left(i\right)}_{BCS}=T(i)b2\;{\left(i\right)}_{LCS}.$$

It means that matrix T(i) transforms LCS of REE to BCS that is a true relation (6). Transformation matrix T(i) is defined by relation [21,41]

(7) T(i)=L(i)⋅Q(i)

where L(i) is translation matrix and Q(i) rotation matrix. The detailed calculation procedure of finding transform matrix T(i) is described in [21,42].

By fulfilling the conditions (6), one possible position of axis $o_{BCS}$ in BCS (and thus the whole frame) is determined during the *i*-th passage of frame through head. $B{\left(j\right)}_{BCS}=T(i)B{\left(j\right)}_{LCS}$ and $B{\left(j\right)}_{BCS}$ $\in o_{BCS}$ is true for 1≤j≤N. The position of $o_{BCS}$ axis is determined by points $B{\left(j\right)}_{BCS}$. At this stage, the best possible position of axis $o_{BCS}$ in BCS is investigated for the needs of the winding process.

Please note that identification $h2\;{\left(i\right)}_{BCS}\equiv b2\;{\left(i\right)}_{BCS}=T(i)b2\;{\left(i\right)}_{LCS}$ in relation (6) enables the possible rotation of the frame around vector $b1\;{\left(i\right)}_{BCS}$ at point $B{\left(i\right)}_{BCS}$.

For each i (1≤i≤N), we seek optimal point $H{\left(i\right)}_{opt_{BCS}}$ and vectors $h1{\left(i\right)}_{opt_{BCS}}$, $h2\;{\left(i\right)}_{opt_{BCS}}$, so that distances ${\left\|P1{\left(i\right)}_{opt_{BCS}}M1_{BCS}\right\|}^{}$ and ${\left\|P2{\left(i\right)}_{opt_{BCS}}M2_{BCS}\right\|}^{}$ are the smallest possible values. It means that point $H{\left(i\right)}_{opt_{BCS}}$ and vectors $h1{\left(i\right)}_{opt_{BCS}}$, $h2{\left(i\right)}_{opt_{BCS}}$ determine the best frame position for the winding process.

Coordinates $x{\left(i\right)}_{opt_{BCS}},\;z{\left(i\right)}_{opt_{BCS}}$ define sought point $H{\left(i\right)}_{{opt}_{BCS}}$ (coordinate $y_{{H\left(i\right)}_{BCS}}$ is constant because $H{\left(i\right)}_{BCS}\in \sigma$), sought unit vectors $h1{\left(i\right)}_{{opt}_{BCS}}$ and $h2{\left(i\right)}_{{opt}_{BCS}}$ are defined by angles $\varphi {\left(i\right)}_{opt}$, $\omega {\left(i\right)}_{opt}$ and by relation (5).

Now, we focus on the definition of cost function F in the form

(8) $$F(i,\;x_{H{\left(i\right)}_{BCS}},\;z_{H{\left(i\right)}_{BCS}},\;\varphi (i),\;\omega (i))=\nu_{1}{\left\|P1{\left(i\right)}_{BCS}M1_{BCS}\right\|}^{2}+\nu_{2}{\left\|P2{\left(i\right)}_{BCS}M2_{BCS}\right\|}^{2},$$

where ${\left\|P1{\left(i\right)}_{BCS}M1_{BCS}\right\|}^{2}={\left(x_{P1{\left(i\right)}_{BCS}}-x_{M1{\left(i\right)}_{BCS}}\right)}^{2}+{\left(y_{P1{\left(i\right)}_{BCS}}-y_{M1{\left(i\right)}_{BCS}}\right)}^{2}+{\left(z_{P1{\left(i\right)}_{BCS}}-z_{M1{\left(i\right)}_{BCS}}\right)}^{2}$,

$${\left\|P2{\left(i\right)}_{BCS}M2_{BCS}\right\|}^{2}={\left(x_{P2{\left(i\right)}_{BCS}}-x_{M2{\left(i\right)}_{BCS}}\right)}^{2}+{\left(y_{P2{\left(i\right)}_{BCS}}-y_{M2{\left(i\right)}_{BCS}}\right)}^{2}+{\left(z_{P2{\left(i\right)}_{BCS}}-z_{M2{\left(i\right)}_{BCS}}\right)}^{2}$$

and $\nu_{1},\;\nu_{2}$ are weight constants which allow the specification of the importance of the first layer and the third layer of winding fibers. It follows from the definition of cost function F, the smaller value of function F, the better winding conditions.

**Note 1**

Ensuring the quality of the third winding layer is often more important than the quality of the first winding layer as it also ensures the fixing of the second placement of fibers in a longitudinal direction (second static guide line provides winding at zero angle). Therefore, constants $\nu_{1},\;\nu_{2}$ can be set $\nu_{2}>\nu_{1}$ in the definition of cost functions F in relation (8).

We find the global minimum of cost function F defined by relation (8) in *i*-th step of passage frame through the fiber-processing head, i.e.,

(9) $$F(i,\;x{\left(i\right)}_{{opt}_{BCS}},\;z{\left(i\right)}_{{opt}_{BCS}},\;\varphi {\left(i\right)}_{opt},\;\omega {\left(i\right)}_{opt})=\underset{x_{H{\left(i\right)}_{BCS}},\;z_{H{\left(i\right)}_{BCS}},\;\varphi (i),\;\omega (i)}{\min}\;\{F(i,\;x_{H{\left(i\right)}_{BCS}},\;z_{H{\left(i\right)}_{BCS}},\;\varphi (i),\;\omega (i))\}$$

Coordinates $x{\left(i\right)}_{{opt}_{BCS}},\;z{\left(i\right)}_{{opt}_{BCS}}$ define sought point $H{\left(i\right)}_{{opt}_{BCS}}$ (coordinate $y_{H{\left(i\right)}_{BCS}}$ is constant), sought unit vectors $h1{\left(i\right)}_{{opt}_{BCS}}$ and $h2{\left(i\right)}_{{opt}_{BCS}}$ are defined by angles $\varphi {\left(i\right)}_{opt}$, $\omega {\left(i\right)}_{opt}$ and by relation (5).

As result of a calculation of $x{\left(i\right)}_{opt},\;z{\left(i\right)}_{opt},\;\varphi {\left(i\right)}_{opt},\;\omega {\left(i\right)}_{opt}$, transformation matrix $T{\left(i\right)}_{opt}$ is obtained.

In accordance with relation (7) the transformation matrix $T{\left(i\right)}_{opt}$ is defined by relation

(10) $$T{\left(i\right)}_{opt}=L{\left(i\right)}_{opt}\cdot Q{\left(i\right)}_{opt}$$

where $L{\left(i\right)}_{opt}$ is the translation matrix and $Q{\left(i\right)}_{opt}$ is the rotation matrix of LCS toward BCS. Rotation matrix $Q{\left(i\right)}_{opt}$ in relation (10) can be decomposed in the form

(11) $$Q{\left(i\right)}_{opt}=Rot(z,a{\left(i\right)}_{opt})\cdot Rot\;(y,b{\left(i\right)}_{opt})\cdot Rot\;(x,c{\left(i\right)}_{opt})$$

where $Rot\;(z,a{\left(i\right)}_{opt})$ is rotation matrix around axis z at angle $a{\left(i\right)}_{opt}$, $Rot\;(y,b\;{\left(i\right)}_{opt})$ is the rotation matrix around axis y at angle $b{\left(i\right)}_{opt}$ and $Rot\;(x,c\;{\left(i\right)}_{opt})$ is the rotation matrix around axis x at angle $c{\left(i\right)}_{opt}$. A detailed procedure of the calculation of Euler angles $a{\left(i\right)}_{opt},\;b{\left(i\right)}_{opt}$ and $c{\left(i\right)}_{opt}$ is described elsewhere [21,42]. Then $TCP{\left(i\right)}_{opt}=(\tilde{x}{\left(i\right)}_{opt},\tilde{y}{\left(i\right)}_{opt},\tilde{z}{\left(i\right)}_{opt},a{\left(i\right)}_{opt},\;b{\left(i\right)}_{opt},\;c\;{\left(i\right)}_{opt})$ where values $\tilde{x}{\left(i\right)}_{opt},\tilde{y}{\left(i\right)}_{opt}$ and $,\tilde{z}{\left(i\right)}_{opt}$ are defined by the elements of matrix $L{\left(i\right)}_{opt}$ (the detail is provided elsewhere [21,42]).

**Note 2**

It is necessary in the *i*-th optimization step to accept only such $TCP\;{\left(i\right)}_{opt}$ whose corresponding parameters with $TCP{\left(i-1\right)}_{opt}$ parameters differ less than the specified limit. If the condition is not satisfied, it is necessary to seek another suitable minimum of cost function F.

Sequence $TCP{\left(i\right)}_{opt}$, 1≤i≤N, is calculated on the external PC and subsequently loaded into the control unit of industrial robot. In this way, the optimal REE trajectory is determined by the procedure described above. Then the control unit of robot interpolates the corresponding parameters of $TCP\;{\left(i\right)}_{opt},$ 1≤i≤N, by its internal interpolation functions similar to what described in [43]. The REE moves according to the off-line optimal trajectory thus determined.

#### 2.3.1. Schematic Representation of the Procedure for Calculating the Optimal REE Trajectory

**Figure 11:**
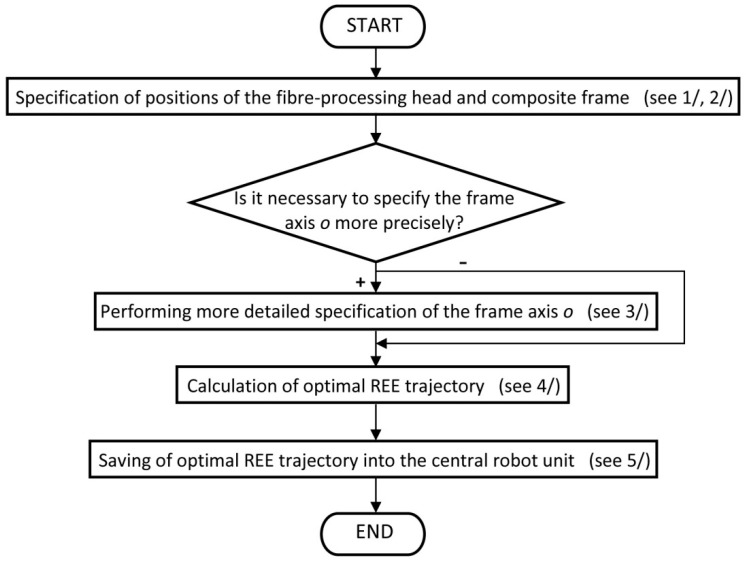
Flowchart of optimal off-line REE trajectory calculation.

The schematic representation of calculation of sequence $TCP{\left(i\right)}_{opt}$ is described in the flowchart as shown in Figure 11.

**Note 3** (based on the flowchart shown in Figure 11)

1. Specification of the fiber-processing head in BCS (including coordinates of centers $S1_{BCS}$ and $S2_{BCS}$ of outer rotating guide lines k1 and k2 of the head, vectors $h1{}_{BCS}$ and $h2{}_{BCS}$, common radius $r_{CIRCLE}$ of circles k1 and k2).
2. Loading of the location of composite frame in LCS (including coordinates of points $B{\left(i\right)}_{LCS}$, vectors $b1\;{\left(i\right)}_{LCS}$, $b2\;{\left(i\right)}_{LCS}$ and values $C{\left(i\right)}_{}$ for 1≤i≤N, radius of frame $r_{TUBE}$).
3. Determination of more $B{\left(i\right)}_{LCS}$ points on frame axis $o_{LCS}$ and corresponding vectors $b1\;{\left(i\right)}_{LCS}$ and $b2\;{\left(i\right)}_{LCS}$.
4. Calculation of the optimal REE trajectory to ensure the high-quality of fiber winding on the composite frame. A differential evolution algorithm (see Section 4) is used for the optimization procedure. Determining the optimal sequence $TCP{\left(i\right)}_{opt}$ (1≤i≤N) is the result of calculation.
5. Storing the calculated sequence of $TCP{\left(i\right)}_{opt}$ (1≤i≤N) in the central robot unit. Determining the optimal trajectory by linking individual corresponding parameters of consecutive following $TCP{\left(i\right)}_{opt}$ (using programming instruction of robot—linear interpolations or cubic splines).

**Figure 12:**
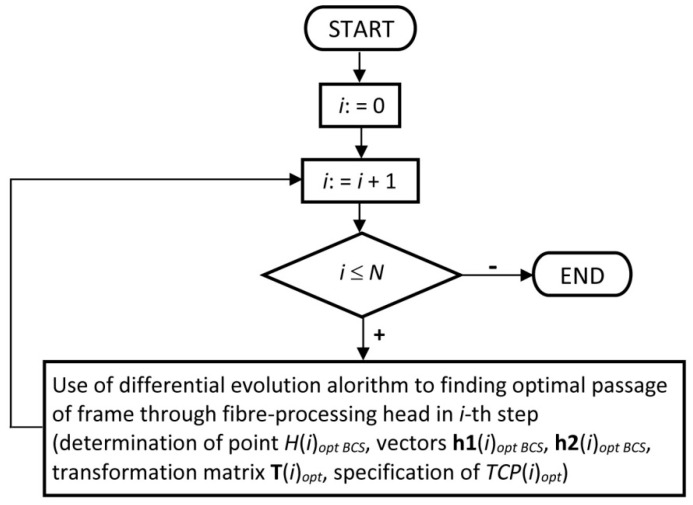
Flowchart of optimal off-line REE trajectory calculation.

The flowchart shown in Figure 12 describes point No. 4 of Notes 3 in more detail—the procedure for calculation of sequence $TCP\;{\left(i\right)}_{opt}$ (1≤i≤N).

**Note 4**

**Figure 13:**
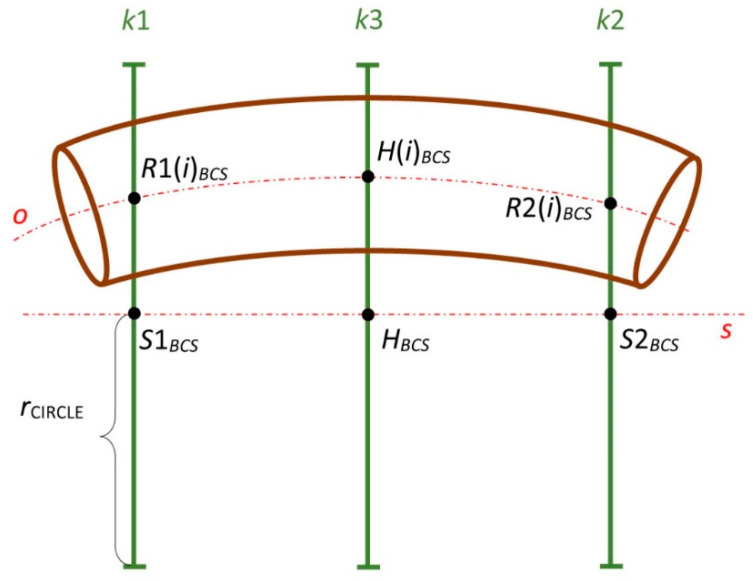
Schematic front view of collision test of non-bearing core frame passage through fiber-processing head (axis $s_{BCS}$ is parallel to axis $y_{BCS}$).

The possible collisions of the composite frame and fiber-processing head (especially collisions of the frame and three guide lines k1, k3 and k2 with common radius $r_{\mathrm{CIRCLE}}$, the radius of frame is $r_{\mathrm{TUBE}}$, where $r_{\mathrm{TUBE}}<r_{\mathrm{CIRCLE}}$, see Figure 13) are tested in each step of passage of the frame through the head.

#### 2.3.2. Use of Differential Evolution Algorithm to REE Trajectory Optimization

The procedure for finding minimum (relation (9)) of cost function F defined by relation (8) in the *i*-th step of frame passage through the winding head, is described. Cost function F often contains many local minima. Thus, using gradient methods for finding the global minimum of function F is not proper (the high probability that is found only local minimum). Genetic algorithm is often applied to finding optimal REE trajectory [30,44]. Hence, a differential evolution algorithm is used to minimize of cost function F. This optimization algorithm gives better results than a genetic algorithm when solving a given minimization problem. Classical differential evolution algorithm is usually denoted DE/rand/1/bin (for more detail see [45,46]). The modified differential algorithm (of DE/rand/1/bin) is used that is hereafter denoted by MDEA [46]. When using MDEA, the asymptotic convergence to the global minimum of cost function F is ensured [46,47]. The use of MDEA has already proven to be successful in optimizing in other technical areas [48]. It is often difficult to find a global minimum of cost function F in the final steps of the algorithm. However, we are then able to find a satisfactory local minimum.

MDEA is used to find $TCP{\left(i\right)}_{opt}$ for given value i, where 1≤i≤N. The position of the frame in BCS is given by four parameters $x_{H{\left(i\right)}_{BCS}},\;z_{H{\left(i\right)}_{BCS}},\;\varphi (i),\;\omega (i)$. These parameters define the value of cost function F specified by relation (8). This means that every four parameters $x_{H{\left(i\right)}_{BCS}},\;z_{H{\left(i\right)}_{BCS}},\;\varphi (i),\;\omega (i)$ define one possible location of the frame in the *i*-th step of passage through the fiber-processing head. One individual y (i) in MDEA is defined by these four parameters and is a potential solution to this problem of finding global minimum (relation (9)) of function F. Specimen SPEC ( i ) is defined and determines the type and value ranges of each parameter of the possible individual y (i) in the *i*-th step of passage of the frame through the fiber-processing head. Then SPEC ( i ) can be expressed in the form [45]

(12) $$SPEC\;(\;i\;)\;=\left\{\left\{\;real,\;Lo_{1}(i),\;Hg_{1}(i)\right\},\;\left\{\;real,\;Lo_{2}(i),\;Hg_{2}(i)\right\},\;\left\{\;real_{3},\;Lo_{3}(i),\;Hg_{3}(i)\right\},\;\left\{\;real,\;Lo_{4}(i),\;Hg_{4}(i)\right\}\right\}$$

Here denomination of real specifies that all four parameters are of a real type, values $Lo_{j}(i)$ and $Hg_{j}(i)$ specify its lower and upper limits, where 1≤j≤4. The specimen determines the admissible parameter values defined location of the frame in BCS.

In the MDEA we successively construct generations of individuals y(i). Each generation includes NP individuals, where each individual y(i) is a potential solution to the problem (relation (9)).

One way of forming the initial generation of individuals y(i) is given by relation

(13) $$y{\left(i\right)}_{m\;,\;j}:=Lo_{j}(i)+\mathrm{rand}\;(0,\;1)\cdot \left(Hg_{j}(i)-Lo_{j}(i)\right)$$

where index i indicates the *i*-th step of optimization, j determines the j-th component of the *m*-th individual of the initial generation (1≤m≤NP, 1≤j≤4). The function rand (0, 1) randomly generates a value from a closed interval <0, 1>.

A sequence of generations G(k) is created, where k denotes the number of the generation. Each generation comprises of individuals y(i) and we look for the individual with the smallest value F(y(i)). In general, four individuals of the current generation of MDEA participate in the creation of an individual of the next generation. The generated individuals are saved in matrix $B\in R^{\mathrm{NP}\;\times \;5}$. Each row of this matrix represents one individual y(i) and its evaluation F(y(i)). During the creation of individuals, it is essential to ensure that the components of each generated individual are consistent with relation (12).

#### 2.3.3. Pseudo-Code of MDEA

In this section, detail information about MDEA is described. The Algorithm 1 consists of three basic parts: necessary input values, its own computational part, and output values of the algorithm. The algorithm is presented in pseudo-code for given fixed number i (determining the *i*-th step of REE trajectory), where 1≤i≤N. The index i is not used in the following algorithm for clarity of written pseudo-code.
**Algorithm 1. MDEA**Input:   The number of calculated generations NG, crossover probability CR, mutation factor f, generation size NP, the dimension of individuals D=4, lower limits $Lo_{j}$ and upper limits $Hg_{j}$, 1≤j≤4. Internal computation: Create an initial generation (k=0) of NP individuals $y_{m}^{k}$, 1≤m≤NP, (e.g., by use of relation (13)).a) Evaluate all the individuals $y_{m}^{k}$ of the *k*-th generation (calculate $F(y_{m}^{k})$ for each individual $y_{m}^{k}$). b) Store the individuals $y_{m}^{k}$ and their evaluations $F(y_{m}^{k})$ into matrix B (each matrix row contains parameters of individual $y_{m}^{k}$ and its evaluation $F(y_{m}^{k})$, 1≤m≤NP).while k≤NG
a) *for*
m:=1
*step*
1 to NP
*do*
 *collision:=true* *repeat*  (i)  randomly select index $s_{m}\in \{1,\;2,\;\ldots \;,\;D\},$  (ii) randomly select indices $r_{1},\;r_{2},\;r_{3}\in \{1,\;\ldots \;,\;NP\},$    where $r_{l}\neq m$ for 1≤l≤3; $r_{1}\neq r_{2},\;r_{1}\neq r_{3},\;r_{2}\neq r_{3}$;  (iii)  *for*
j:=1
*step*
1 to D
*do*
     *if*
rand (0, 1)
≤CR or $j=s_{m}$) *then*      $y_{m,j}^{trial}:=y_{r_{3},j}^{k}+f\;\left(y_{r_{1},j}^{k}-y_{r_{2},j}^{k}\right)$                   *else*
      $y_{m,j}^{trial}:=y_{m,j}^{k}$
     *end for (j)*  (iv) Testing of possible collisions of the frame location in BCS defined by $y_{m}^{trial}$    and the fiber-processing head.    *if*
$y_{m}^{trial}$ does not include collisions *then*     *collision* = *false* *until collision* = *false* *end repeat*  (v) *if*
$F\left(y_{m}^{trial}\right)\leq F\left(y_{m}^{k}\right)$
*then*
$y_{m}^{k+1}:=y_{m}^{trial}$              *else*
$y_{m}^{k+1}:=y_{m}^{k}$    *end if* *end for (m)* b) Store individuals $y_{m}^{k+1}$ and their evolutions $F\left(y_{m}^{k+1}\right)$
(1≤m≤NP) of the new generation (k+1)-st generation in the matrix B, k:=k+1. c) Find index p which satisfies the condition $F\left(y_{p}^{k+1}\right)\geq F(y_{m}^{k})$ for 1≤m≤NP, $y_{p}^{k}:=y^{rand}$, where $y^{rand}$ satisfies (13) *end while* (*k*). **Output:** The best found individual $y_{opt}$ is represented by the row of matrix B that contains the corresponding value $\min\{F\left(y_{m}^{k}\right)\;;\;y_{m}^{k}\in B\}$. **Comments.** The *repeat until condition cycle* is always executed at least once, since the controlling condition is checked at the end of the cycle.     Function rand (0, 1) randomly picks a number from the interval 〈0, 1〉. The notation $y_{m,j}^{k}$ means the *j*-th component of an individual $y_{m}^{k}$ in the *k*-th generation. The individual $y_{opt}$ in pseudo-code of MDEA is the final solution and corresponds to designation $y{\left(i\right)}_{opt}$ that includes optimized parameters $x_{H{\left(i\right)}_{opt}},\;z_{{H(i)}_{opt}},\;\varphi {\left(i\right)}_{opt}$, $\omega {\left(i\right)}_{optn}$. However, it should be noted that in general parameters $x_{H{\left(i\right)}_{opt}},\;z_{{H(i)}_{opt}},\;\varphi {\left(i\right)}_{opt}$, $\omega {\left(i\right)}_{opt}$ calculated by MDEA can only be optimized (and not optimal) parameters in relation to equation (9). This is due to the calculation of the final number of generations of individuals using MDEA. Therefore we mark calculated parameters as optimal parameters $x_{H{\left(i\right)}_{opt}},\;z_{{H(i)}_{opt}},\;\varphi {\left(i\right)}_{opt},\;\omega {\left(i\right)}_{opt}$.

## 3. Mechanical Performance of Polymer Composite Frame

**Figure 14:**
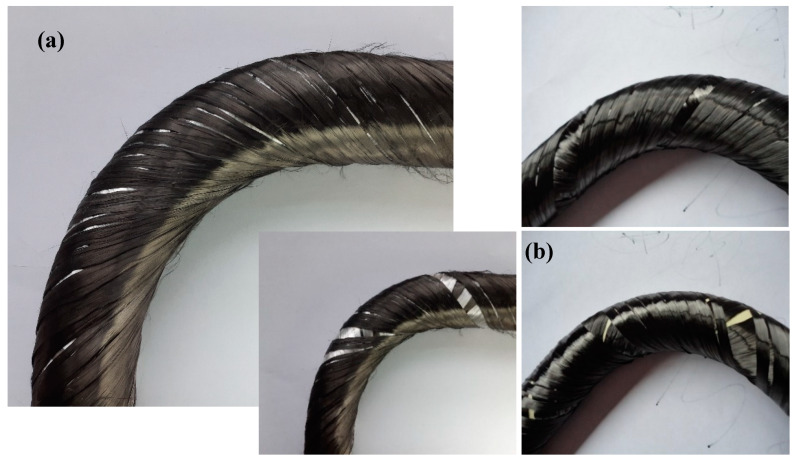
Image of the polymer composite frames wound using (**a**) the new and (**b**) manual-robot winding processes.

The polymer composite frames are designed and fabricated to sustain various loads under specific boundary condition. In this regard, the frame would normally face different types of tensile, compressive, shear, bending and twisting loads. Such a complex loading normally results in the development of various stresses in the composite material and cause early damage phenomena. On the other hand, the composite frames with the complex geometry in open or close form, by nature, are under stress concentration phenomena, which normally are intensified at the curved location of the frame. As mentioned in this study, one of the technological challenges in composite frame production is the homogenous fiber winding process especially at the curved section of the frame. In this regard, few images of the final winding product (before matrix injection) of an L-shape frame based on the manual- and new-robot winding processes are provided, as shown in Figure 14. Results of the new winding method indicated that the angle of the fibers to the frame remains uniform, homogeneous, and consistent in circular-helix cross-sectional at the critical curved section, while the manual-robot winding resulted in random inconsistent fiber winding at different plies. Therefore, the final product of the composite frame using manual-robot winding would contain large macro-scale sections of polymer matrix pocket (with no fiber) at various layers in the curved location where is under stress concentration. In fact, due to the superb mechanical properties of the fiber materials, the existence of matrix pocket itself, would act as a location for stress concentration, even if it was made at the straight locations of the frame that extensively weaken the composite frame [6,36,49,50].

Damage phenomena in normal composite structures (with uniform, homogeneous, and consistent fiber arrangement) under loading normally initiate with micro-scale matrix cracking/crushing that grow in size across the ply thickness (parallel to the fibers), and induce early stage of multi-delamination event. Further loading could result in local fiber cracking/buckling, excessive multiple intra- and inter-laminar failures, weaken the laminate and lead to structural rupture [13,51,52,53,54]. The fiber arrangement in normal composites would result the fibers as the load-bearing core component of the composite, to sustain the load, and ensure the composite frame to bear the designed load before fracture. The process of damage in normal composites occurs gradually, and composite structures are normally able to sustain severe loading conditions [54,55]. However, due to the inconsistent fiber arrangement in composite frame made by manual-robot winding, and stress concentration phenomena at curved sections as well as the matrix pocket parts, the material damage that initiate as matrix micro-crack would easily shift to cross-sectional fracture of the frame at the matrix pocket locations. In this condition, due to the very low properties of matrix materials in comparison with the fiber properties (10%), it is expected that each ply could only sustain about 10% of the load-share assumed for the composite ply. This excessively diminish the yielding limit and desired tolerance of the composite frame [6,53,55,56].

## 4. Practical Experimental Verification Tests of Optimization Procedure

The calculation method of the optimal REE trajectory described in this article was applied to practical problems. If the non-bearing core frame is not too complicated a 3D shape then it is possible to use a simpler non-optimized algorithm to calculate the REE trajectory described by Martinec, et al. [21]. For more complicated 2D and 3D shaped frames it is suitable to use the algorithm for calculating of optimal REE trajectory described in this article.

Entering of the input data is simple and as the output values of the described procedure we get the resulting sequence $TCP{\left(i\right)}_{opt}$, 1≤i≤N. In this way, optimal REE trajectory is calculated on an external PC. Sequence $TCP{\left(i\right)}_{opt}$ is subsequently stored in the central robot unit and in this way optimal REE trajectory is defined.

The focus was on calculation of optimized REE trajectory for two non-bearing core frames with a circular cross-section. In the first experimental test, a normal 2D shape was selected, while a very complicated 3D shape was selected for the second experiment. Cost function F defined by relation (8) is used in the optimization procedure of both cases.

The three layers of the fibers are simultaneously wound on the frame at angles 45°, 0° and −45° and parameters $\nu_{1}=\nu_{2}=1$ in definition of cost function F as provided in relation (8).

### 4.1. Experimental Test 1—Composite Non-Bearing Core Frame Shaped in 2D

**Figure 15:**
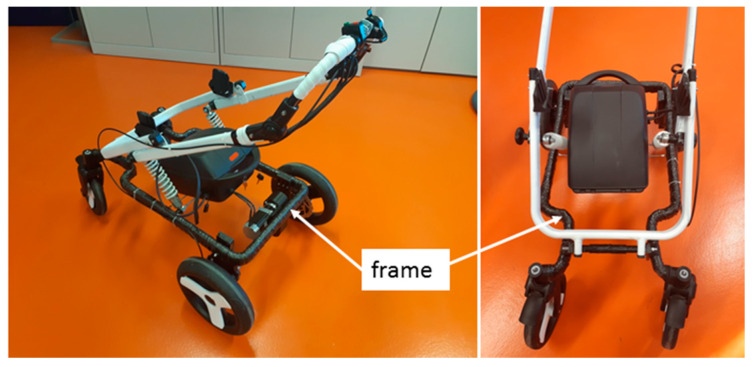
Test 1—Prototype of the baby carriage with supporting composite frame.

The considered composite frame is part of the supporting structure of a baby carriage prototype (Figure 15).

**Figure 16:**
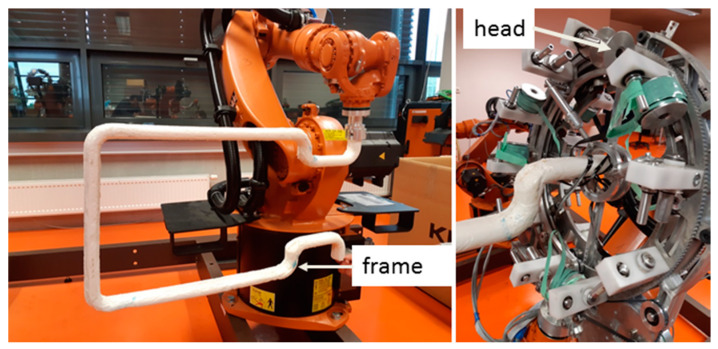
Test 1—Non-bearing core frame connected to end-effector of industrial robot KUKA KR 16-2 (**left**). Testing of passage of the frame through fiber-processing head (**right**).

The non-bearing core frame before fiber winding is shown in Figure 16 (left). The fiber-processing head (Figure 16 (right)) is represented in the model by circle k1 and k2 (they correspond to outer guide lines, see Figure 7 on the left) with centers $S1_{BCS}=$ [1000, 300, 1400, 1] and $S2_{BCS}=$ [1000, 370, 1400, 1] with radius $r_{\mathrm{CIRCLE}}=$ 50 [mm]. The length of straight line segment $S1_{BCS}S2_{BCS}=$ 70 [mm]. It is also valid that $h1_{BCS}\;=(S2_{BCS}-S1_{BCS})/\left\|S2_{BCS}-S1_{BCS}\right\|=(0,\;1,\;0,\;0)$,$h2_{BCS}=\left(0,\;0,\;1,\;0\right)$, $H_{BCS}=\;(S1_{BCS}+S2_{BCS})/2$.

**Figure 17:**
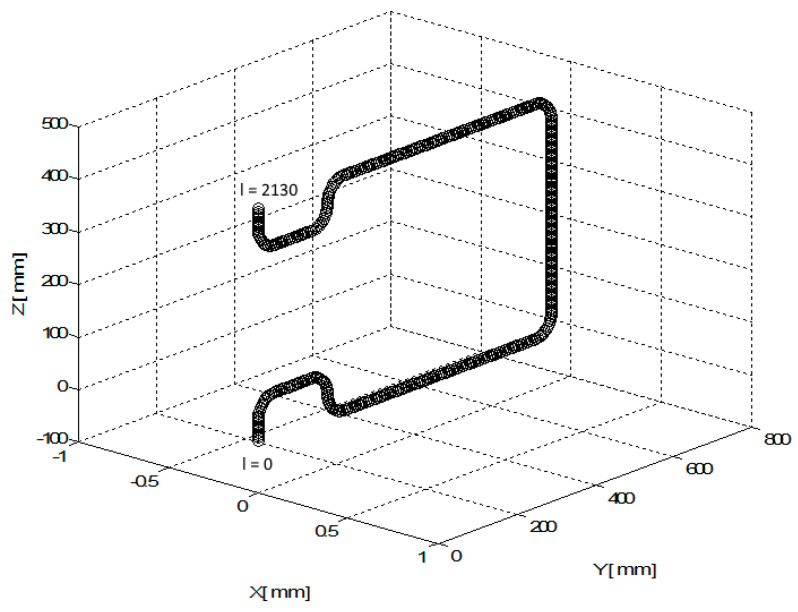
Test 1—3D view of the non-bearing core frame location in LCS.

The vertical cross-section of the frame with axis $o_{LCS}$ and the describing values mentioned in LCS are shown in Figure 8. The position of the frame in LCS in a 3D view is shown in Figure 17.

Axis $o_{LCS}$ of the composite frame is entered in LCS of the REE using the discrete set of points $B(i){}_{LCS}$, 1≤i≤N=2131. The total length of axis $o_{LCS}$ (o-arc length) from the starting point $B{\left(1\right)}_{LCS}$ to the end point $B{\left(N\right)}_{LCS}$ is C(N)=2130  [mm]. The continuous distance of points $B(i){}_{LCS}$ on axis $o_{LCS}$ from the initial point $B{\left(1\right)}_{LCS}$ is denoted by C(i), 1≤i≤N=2131. Vectors $b1\;(i){}_{LCS}$ and $b2\;{\left(i\right)}_{\;LCS}$ are specified and the radius of the frame is $r_{\mathrm{TUBE}}=$ 20 [mm] (the frame has the same cross-section on the whole circuit of the frame).

**Figure 18:**
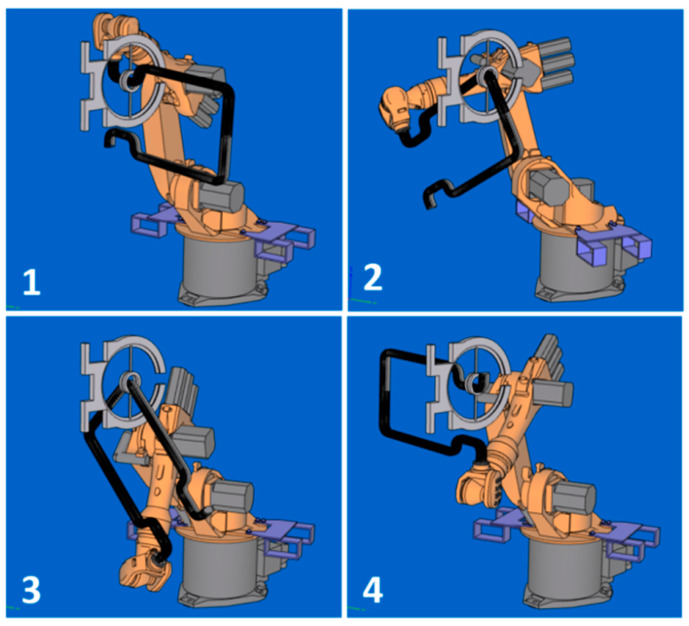
Test 1—the graphical simulation of the robot position and the frame in BCS in the winding at four selected points (**1**–**4**) of optimal trajectory.

The calculated optimal REE trajectory was first used for graphical simulation of the frame passage through the fiber-processing head using the OfficeLite simulator software and the KUKASimPro graphic simulator of the actions of the robot (see Figure 18). Optimal REE trajectory was subsequently tested in a composite experimental workplace.

**Figure 19:**
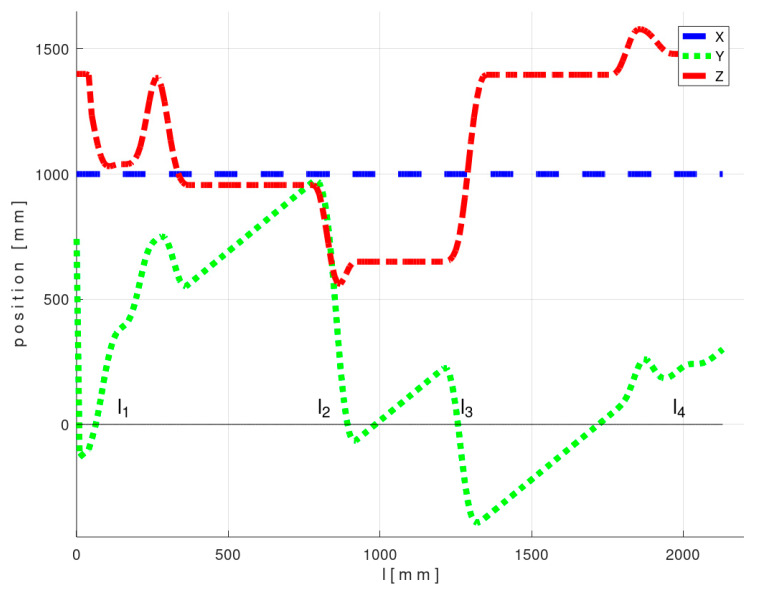
Test 1—Diagram of the optimal course of the $TCP_{OPT}$ during the passage of the frame through the fiber-processing head-values of the first three parameters of optimal $TCP_{OPT}$.

**Figure 20:**
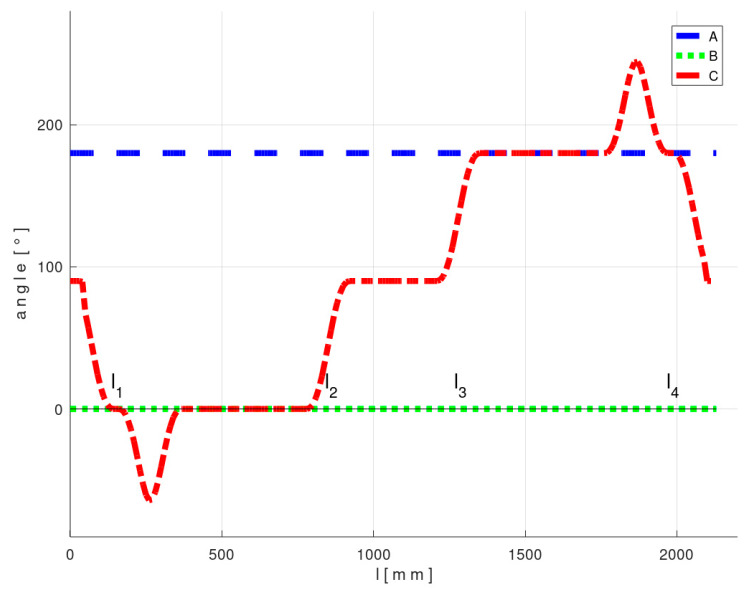
Test 1—Diagram of the optimal course of the $TCP_{OPT}$ during the passage of the frame through the fiber-processing head - values of the last three parameters of optimal $TCP_{OPT}$.

The course of the REE and its location in BCS during the passage of the frame through the fiber-processing head is graphically illustrated in Figure 19 and Figure 20. The $l_{1},\;l_{2},\;l_{3}$ and $l_{4}$ values in Figure 19 and Figure 20 in the l-axis correspond to positions of REE in sub-figures 1, 3, 4 and 5 in Figure 18.

The calculated optimized trajectory lies in a plane parallel to axes *z* and *y* of BCS. Therefore, parameters *x*, *a*, and *b* in optimized $TCP_{OPT}$ are constant for the entire optimal trajectory.

**Figure 21:**
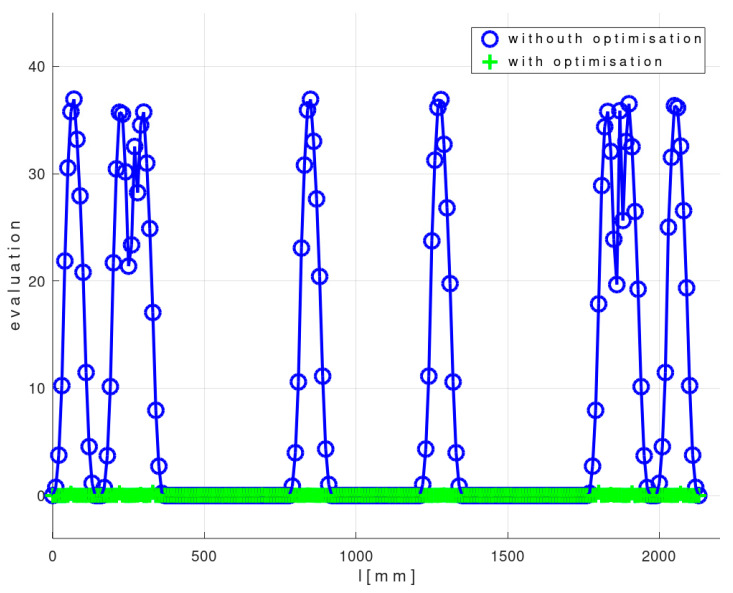
Test 1—Diagram showing the course of function $F(x{\left(i\right)}_{opt},\;z{\left(i\right)}_{opt},\;\varphi {\left(i\right)}_{opt},\;\omega {\left(i\right)}_{opt})$ for optimal REE trajectory and values $F(x_{H_{BCS}},\;z_{H_{BCS}},\;0,\;0)$ for non-optimal REE trajectory during the passage of the frame through the fiber-processing head (1≤i≤N=2131).

Figure 21 shows a graph of the optimal values $F(x{\left(i\right)}_{opt},\;z{\left(i\right)}_{opt},\;\varphi {\left(i\right)}_{opt},\;\omega {\left(i\right)}_{opt})$ (see relation (9)) corresponding to the optimal trajectory REE and a graph of values $F(x_{H_{BCS}},\;z_{H_{BCS}},\;0,\;0)$ corresponding to the non-optimized REE trajectory described in [21].

### 4.2. Experimental Test 2—3D Shape Non-Bearing Core Frame

**Figure 22:**
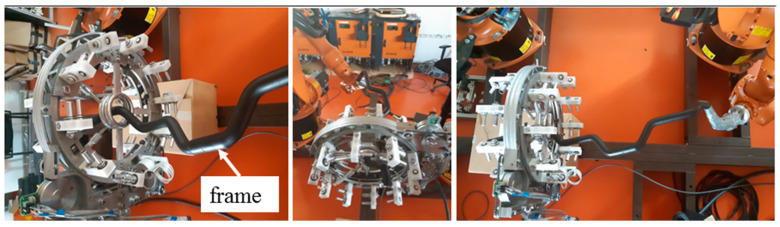
Test 2—Frame passage testing through fiber-processing head for calculated optimal REE trajectory.

In the second practical example, the calculation of optimal REE trajectory is performed for a 3D shaped frame shown in Figure 1 and Figure 22 (on the left and on the right). After production, this composite frame serves to reinforce the car chassis.

The fiber-processing head is defined by the same parameter values as in the previous experimental test No. 1, but only $S1_{BCS}=$ [400, 1000, 1600, 1] and $S2_{BCS}=$ [400, 1070, 1600, 1].

Axis $o_{LCS}$ of the non-bearing core frame is entered in LCS of the REE by a discrete set of points $B(i){}_{LCS}$, 1≤i≤N=1031. The total length of axis $o_{LCS}$ (o-arc length) from the starting point $B{\left(1\right)}_{LCS}$ to the end point $B\;(N){}_{LCS}$ is C (N)=1030 [mm] (note that continuous distance of points $B(i){}_{LCS}$ on axis $o_{LCS}$ from the initial point $B{\left(1\right)}_{LCS}$ is denoted by C(i)). Vectors $b1\;(i){}_{LCS}$ and $b2\;{\left(i\right)}_{\;LCS}$ are specified and the radius of the frame is $r_{\mathrm{TUBE}}=20\;$ [mm] (the frame has the same radius of cross-section on the whole circuit of the frame).

**Figure 23:**
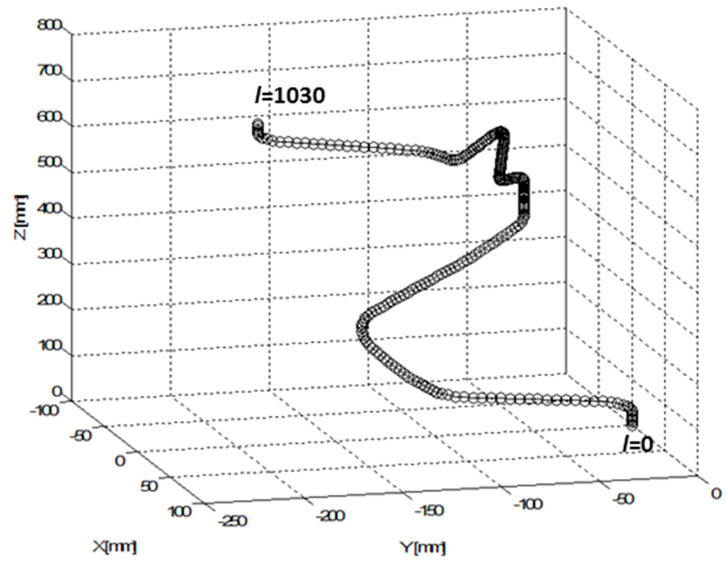
Test 2—3D graphical representation of axis $o_{LCS}$ in LCS.

A graphical representation of the specified axis $o_{LCS}$ of the frame defined in LCS is shown in Figure 23. Initial point $B{\left(1\right)}_{BCS}$ corresponds to l=  *C*(1) = 0 [mm] and trajectory endpoint $B{\left(1031\;\right)}_{BCS}$ corresponds to l=C (1031)=1030 [mm].

**Figure 24:**
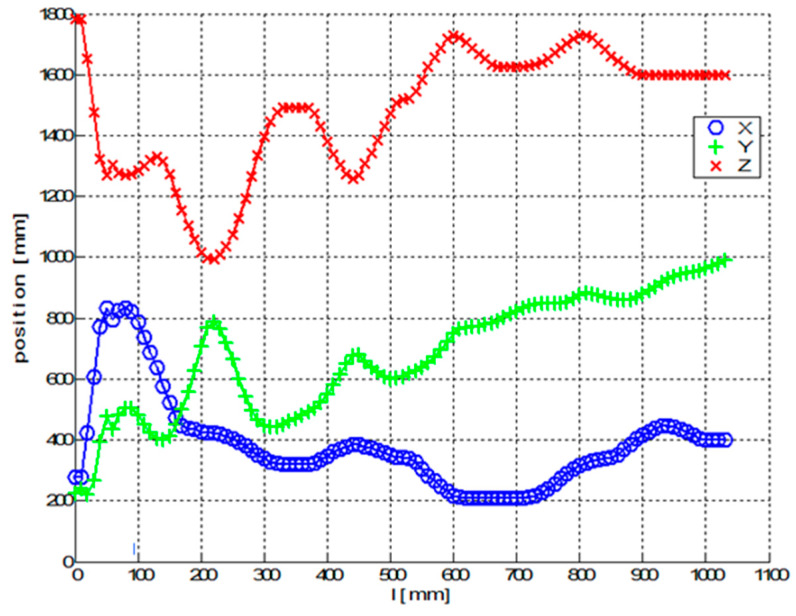
Test 2—Diagram of the optimal course of the $TCP_{OPT}$ during the passage of the frame through the fiber-processing head-values of the first three parameters of $TCP_{OPT}$.

**Figure 25:**
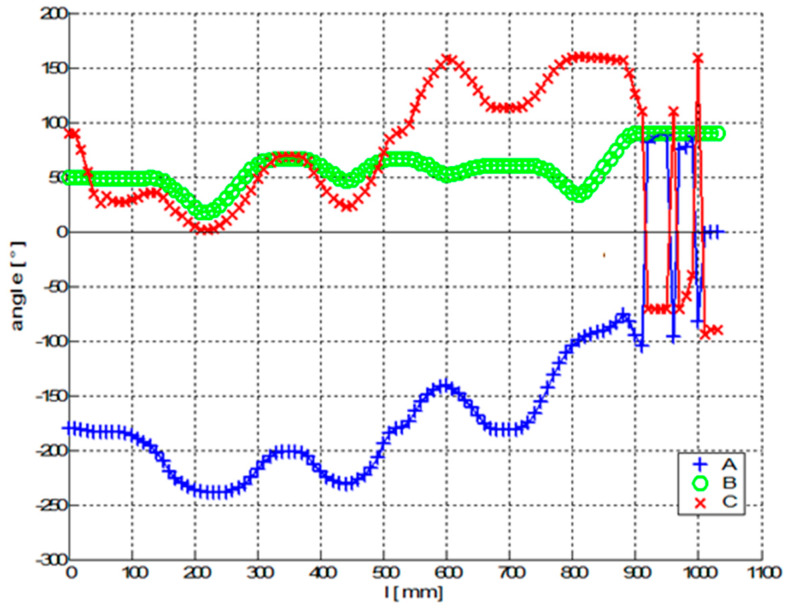
Test 2—Diagram of the optimal course of the $TCP_{opt}$ during the passage of the frame through the fiber-processing head—values of the last three parameters of $TCP_{opt}$.

Figure 24 and Figure 25 show graphs of parameters of optimal $TCP_{OPT}$ during the winding of fibers on the frame. The graphs in Figure 24 contain the first three parameters and the graphs in Figure 25 show the last three parameters of optimal $TCP_{OPT}$.

Unlike the previous experimental Test 1, the frame is 3D shaped and all six parameters of optimized $TCP_{OPT}$ change continuously during the winding process. The sudden changes in a and b parameters in Figure 25 do not affect the rapid changes in REE orientation. It should be noted that orientation of the REE is defined by rotation matrix $Q{\left(i\right)}_{opt}$ in relation (10). The determination of Euler angles *a*, *b* and *c* are not unique [21,57]. Thus, in general, different parameter values can give the same matrix $Q{\left(i\right)}_{opt}$ in the product of sub-rotation matrices (relation (11)).

**Figure 26:**
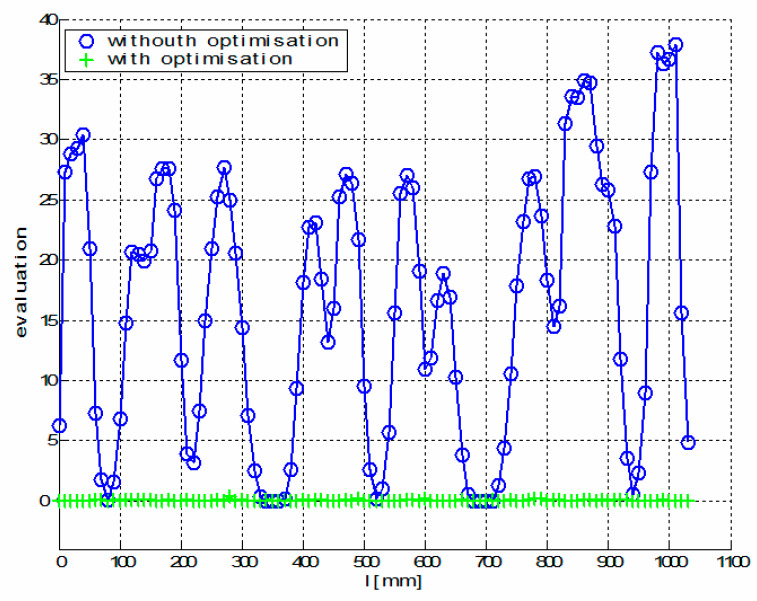
Experimental Test 2—Diagram showing the course of function $F(x{\left(i\right)}_{opt},\;z{\left(i\right)}_{opt},\;\varphi {\left(i\right)}_{opt},\;\omega {\left(i\right)}_{opt})$ for optimal REE trajectory and values $F(x_{H_{BCS}},\;z_{H_{BCS}},\;0,\;0)$ for non-optimal REE trajectory during the passage of the frame through the fiber-processing head (1≤i≤N=1031).

Figure 26 shows a graph of the optimal values $F(x{\left(i\right)}_{opt},\;z{\left(i\right)}_{opt},\;\varphi {\left(i\right)}_{opt},\;\omega {\left(i\right)}_{opt})$ (see relation (9)) corresponding to the optimal trajectory REE and a graph of values $F(x_{H_{BCS}},\;z_{H_{BCS}},\;0,\;0)$ corresponding to the non-optimized REE trajectory [21].

It follows from values of cost function F defined by relation (8) in Figure 26 (and also in Test 1—Figure 21) that the optimized calculation method for definition of REE trajectory is more suitable than the simple non-optimized method defined in [21] for complex frame shapes.

## 5. Conclusions

Fabrication of high-quality polymer composite frame is highly dependent on the homogeneous and uniform winding process of filament fibers. In the dry fiber winding technology on a non-bearing core frame, three layers of fibers are gradually created on the frame. The quality of each fiber winding layer depends on keeping the correct winding angle and thereby ensuring the homogeneity of the wound fibers. Fulfillment of these conditions depends on determining the correct REE trajectory, especially in the case of a 3D shaped frame. The simple calculation method of an off-line REE trajectory described in [21] is suitable only for frames that do not have an excessively complicated shape.

In this study, the original calculation method of optimal REE trajectory is developed, described and experimentally tested to ensure optimal possible correct winding angles of individual fiber layers onto frame. This method provides a significant advantage over a manually entered REE trajectory (manual-robot winding). Technicians often prefer the manual setting of the REE trajectory by means of a teach pendant (control body for motions of a robot [58]). However, this approach of trajectory determination is time-consuming and in practice it is not feasible to define an optimal REE trajectory for our needs.

Using the above described optimization algorithm, is appropriate in general for any shape of frame with a circular cross-section, especially in the case of a more complicated 3D frame shape. The use of the described procedure makes it possible to ensure the desired fiber angles and hence the homogeneity of the fiber windings. Such a process makes it possible to produce a composite frame with high resistance to operational stress. As a result, the mechanical performance of composite frame could be assumed based on the designed load, while the load capacity of the composite frame made of manual-robot winding is diminished drastically.

The principle of the optimization algorithm can also be applied to other manufacturing production of specific composites when it is necessary to determine the 3D trajectory of the REE.

The described optimization algorithm is completely independent of the type of the industrial robot and the robot software tool used. The optimization algorithm can be used successfully by technicians of industrial composite workplaces and also by programmers of software tools for industrial robots involved in production of composites.

At present, specialized companies offer commercial software tools to industrial robot users. These tools are developed for advanced areas of industrial production (e.g., cutting, welding, pressing, or packing). However, these tools are not usually available for special production technologies such as the manufacturing of composite frames and other types of composites [59,60]. In such cases, the described optimization algorithm for calculation of an optimal REE trajectory can be used successfully.

## References

[1] Gay D. (2014). Composite Materials: Design and Applications.

[2] Koloor S.S.R., Tamin M. (2018). Mode-II interlaminar fracture and crack-jump phenomenon in CFRP composite laminate materials. Compos. Struct..

[3] Mlynek J., Petru M., Martinec T. Optimization of Industrial Robot Trajectory in Composite Production. Proceedings of the 2018 18th International Conference on Mechatronics-Mechatronika (ME).

[4] Petrů M., Mlynek J., Martinec T. (2018). Numerical modelling for optimization of fibres winding process of manufacturing technology for the non-circular aerospaces frames. Manuf. Technol..

[5] Kulhavy P., Syrovatkova M., Srb P., Petru M., Samkova A. (2017). Irregular Winding of Pre-preg Fibres Aimed at the Local Improvement of Flexural Properties. Tekstilec.

[6] Koloor S.S.R., Khosravani M.R., Hamzah R., Tamin M. (2018). FE model-based construction and progressive damage processes of FRP composite laminates with different manufacturing processes. Int. J. Mech. Sci..

[7] Wang X., Petrů M., Yu H. (2019). The effect of surface treatment on the creep behavior of flax fiber reinforced composites under hygrothermal aging conditions. Constr. Build. Mater..

[8] Sharma S., Sowntharya L., Kar K.K. (2017). Polymer-Based Composite Structures: Processing and Applications. Composite Materials.

[9] Agarwal B.D., Broutman L.J., Chandrashekhara K. (2017). Analysis and Performance of Fiber Composites.

[10] Sharifi Teshnizi S.H., Koloor S.S.R., Sharifishourabi G., Bin Ayob A., Yahya M.Y. (2012). Effect of ply thickness on displacements and stresses in laminated GFRP cylinder subjected to radial load. Adv. Mater. Res..

[11] Curliss D.B., Lincoln J.E. (2016). Fiber Winding System for Composite Projectile Barrel Structure. U.S. Patent.

[12] Hernandez-Moreno H., Douchin B., Collombet F., Choqueuse D., Davies P. (2008). Influence of winding pattern on the mechanical behavior of filament wound composite cylinders under external pressure. Compos. Sci. Technol..

[13] Fowler C.P., Orifici A.C., Wang C.H. (2016). A review of toroidal composite pressure vessel optimisation and damage tolerant design for high pressure gaseous fuel storage. Int. J. Hydrog. Energy.

[14] McIlhagger A., Archer E., McIlhagger R. (2020). Manufacturing processes for composite materials and components for aerospace applications. Polymer Composites in the Aerospace Industry.

[15] Groppe D. (2000). Robots improve the quality and cost-effectiveness of composite structures. Ind. Robot.

[16] Mlýnek J., Petrů M., Martinec T. Design of composite frames used in agricultural machinery. Proceedings of the 7th TAE.

[17] Koloor S., Abdullah M., Tamin M., Ayatollahi M. (2019). Fatigue damage of cohesive interfaces in fiber-reinforced polymer composite laminates. Compos. Sci. Technol..

[18] Quanjin M., Rejab M., Idris M., Kumar N.M., Merzuki M. (2018). Robotic Filament Winding Technique (RFWT) in Industrial Application: A Review of State of the Art and Future Perspectives. Int. Res. J. Eng. Technol..

[19] Shirinzadeh B., Alici G., Foong C.W., Cassidy G. (2004). Fabrication process of open surfaces by robotic fibre placement. Robot. Comput.-Integr. Manuf..

[20] Meng Z., Yao L., Bu J., Sun Y. (2019). Prediction method for offset compensation on three-dimensional mandrel with spatial irregular shape. J. Ind. Text..

[21] Martinec T., Mlýnek J., Petrů M. (2015). Calculation of the robot trajectory for the optimum directional orientation of fibre placement in the manufacture of composite profile frames. Robot. Comput.-Integr. Manuf..

[22] Sofi T., Neunkirchen S., Schledjewski R. (2018). Path calculation, technology and opportunities in dry fiber winding: A review. Adv. Manuf. Polym. Compos. Sci..

[23] Polini W., Sorrentino L. (2005). Influence of winding speed and winding trajectory on tension in robotized filament winding of full section parts. Compos. Sci. Technol..

[24] Azevedo C.B., Almeida J.H.S., Flores H.F., Eggers F., Amico S.C. (2020). Influence of mosaic pattern on hygrothermally-aged filament wound composite cylinders under axial compression. J. Compos. Mater..

[25] Gao J., Pashkevich A., Caro S. (2017). Manipulator motion planning in redundant robotic system for fiber placement process. New Trends in Mechanism and Machine Science.

[26] Chen X., Zhang Y., Xie J., Du P., Chen L. (2018). Robot needle-punching path planning for complex surface preforms. Robot. Comput.-Integr. Manuf..

[27] Andulkar M.V., Chiddarwar S.S. (2015). Incremental approach for trajectory generation of spray painting robot. Ind. Robot.

[28] Gao J., Pashkevich A., Caro S. (2017). Optimization of the robot and positioner motion in a redundant fiber placement workcell. Mech. Mach. Theory.

[29] Xiao Y., Du Z., Dong W. (2012). Smooth and near time-optimal trajectory planning of industrial robots for online applications. Ind. Robot.

[30] Piao S., Zhong Q., Wang X., Gao C. Optimal Trajectory Generation for Soccer Robot Based on Genetic Algorithms. Proceedings of the International Workshop on Computer Science for Environmental Engineering and EcoInformatics.

[31] Chen Y., Yan L., Wei H., Wang T. (2013). Optimal trajectory planning for industrial robots using harmony search algorithm. Ind. Robot.

[32] Simba K.R., Uchiyama N., Sano S. (2016). Real-time smooth trajectory generation for nonholonomic mobile robots using Bézier curves. Robot. Comput.-Integr. Manuf..

[33] Hodgkinson J.M. (2000). Mechanical Testing of Advanced Fibre Composites.

[34] Gay D., Gambelin J. (2008). Structural Modelling and Calculus: An Introduction.

[35] Koloor S.S.R., Abdul-Latif A., Tamin M.N. (2011). Mechanics of composite delamination under flexural loading. Key Eng. Mater..

[36] Koloor S.S.R., Hussin H., Tamin M.N. (2012). Mode i interlaminar fracture characterization of CFRP composite laminates. Adv. Mater. Res..

[37] Sharifi Teshnizi S.H., Koloor S.S.R., Sharifishourabi G., Bin Ayob A., Yahya M.Y. (2012). Mechanical behavior of GFRP laminated composite pipe subjected to uniform radial patch load. Adv. Mater. Res..

[38] Sciavicco L., Siciliano B. (2012). Modelling and Control of Robot Manipulators.

[39] Rao J.S., Dukkipati R.V. (1989). Mechanism and Machine Theory.

[40] Budinský B. (1983). Analytic and Differential Geometry. Mathematics for Technical Colleges.

[41] Jazar R.N. (2010). Theory of Applied Robotics: Kinematics, Dynamics, and Control.

[42] Mlýnek J., Martinec T. Mathematical model of composite manufacture and calculation of robot trajectory. Proceedings of the 16th International Conference on Mechatronics-Mechatronika 2014.

[43] Antia H.M. (2002). Numerical Methods for Scientists and Engineers.

[44] Tian L., Collins C. (2004). An effective robot trajectory planning method using a genetic algorithm. Mechatronics.

[45] Price K., Storn R.M., Lampinen J.A. (2006). Differential Evolution: A Practical Approach to Global Optimization.

[46] Knobloch R., Mlýnek J., Srb R. (2017). The classic differential evolution algorithm and its convergence properties. Appl. Math..

[47] Hu Z., Xiong S., Su Q., Zhang X. (2013). Sufficient conditions for global convergence of differential evolution algorithm. J. Appl. Math..

[48] Mlýnek J., Knobloch R. (2018). Model of shell metal mould heating in the automotive industry. Appl. Math..

[49] Abdi B., Koloor S.S.R., Abdullah M.R., Ayob A., Yahya M.Y.B. (2012). Effect of strain-rate on flexural behavior of composite sandwich panel. Appl. Mech. Mater..

[50] Koloor S.S.R., Tamin M.N. Effects of lamina damages on flexural stiffness of CFRP composites. Proceedings of the 8th Asian-Australasian Conference on Composite Materials 2012, ACCM 2012—Composites: Enabling Tomorrow’s Industry Today.

[51] Schuecker C., Pettermann H. (2008). Fiber reinforced laminates: Progressive damage modeling based on failure mechanisms. Arch. Comput. Methods Eng..

[52] Xian G., Wang Z., Beaumont, Peter W.R., Zweben, Carl H. (2000). Carbon Fiber Reinforced Plastics—Properties. Comprehensive Composite Materials.

[53] Hallett S.R., Jiang W.-G., Khan B., Wisnom M.R. (2008). Modelling the interaction between matrix cracks and delamination damage in scaled quasi-isotropic specimens. Compos. Sci. Technol..

[54] Maimi P., Camanho P., Mayugo J., Turon A. (2011). Matrix cracking and delamination in laminated composites. Part II: Evolution of crack density and delamination. Mech. Mater..

[55] Koloor S.S.R., Karimzadeh A., Yidris N., Petrů M., Ayatollahi M.R., Tamin M.N. (2020). An energy-based concept for yielding of multidirectional FRP composite structures using a mesoscale lamina damage model. Polymers.

[56] Koloor S., Ayatollahi M., Tamin M. (2017). Elastic-damage deformation response of fiber-reinforced polymer composite laminates with lamina interfaces. J. Reinf. Plast. Compos..

[57] Slabaugh G.G. (1999). Computing Euler angles from a rotation matrix. Retr. August.

[58] Brunete A., Mateo C., Gambao E., Hernando M., Koskinen J., Ahola J.M., Seppälä T., Heikkila T. (2016). User-friendly task level programming based on an online walk-through teaching approach. Ind. Robot.

[59] Luo Z. (2015). Robotics, Automation, and Control in Industrial and Service Settings.

[60] Klimchik A., Ambiehl A., Garnier S., Furet B., Pashkevich A. (2017). Efficiency evaluation of robots in machining applications using industrial performance measure. Robot. Comput.-Integr. Manuf..

